# Comparative multi “omics” profiling of *Gossypium hirsutum* and *Gossypium barbadense* fibers at high temporal resolution reveals key differences in polysaccharide composition and associated glycosyltransferases

**DOI:** 10.3389/fpls.2026.1639424

**Published:** 2026-02-19

**Authors:** Sivakumar Swaminathan, Youngwoo Lee, Corrinne E. Grover, Megan F. DeTemple, Alither S. Mugisha, Lauren E. Sichterman, Pengcheng Yang, Jun Xie, Jonathan F. Wendel, Daniel B. Szymanski, Olga A. Zabotina

**Affiliations:** 1 Roy J Carver Department of Biochemistry, Biophysics and Molecular Biology, Iowa State University, Ames, IA, United States; 2 Department of Botany and Plant Pathology, Purdue University, West Lafayette, IN, United States; 3 Department of Ecology, Evolution and Organismal Biology, Iowa State University, Ames, IA, United States; 4 Department of Statistics, Purdue University, West Lafayette, IN, United States

**Keywords:** cellulose, cotton, fiber, glycosyltransferases, hemicelluloses, pectins, proteome, transcriptome

## Abstract

Among the two allopolyploid cultivated species of cotton, *Gossypium barbadense* is known for its superior quality fiber compared to *Gossypium hirsutum*. Length and strength are key determinants of the fiber quality. Although mature fibers are composed of dried cell walls that mainly consist of cellulose, the dynamic remodeling of pectin, xyloglucan, and xylan polysaccharides during fiber growth significantly impacts the final fiber quality. Comprehensive knowledge of polysaccharides and their biosynthesis during fiber development in cultivated species is crucial for improving fiber quality. In this study, comparative large-scale glycome, transcriptome and proteome profiling were conducted daily on fibers of both cotton species, covering critical stages of fiber development spanning primary cell wall synthesis and the transition to secondary cell wall synthesis. Interspecific comparisons revealed that a delayed deposition of cellulose content, as well as the occurrence of lower levels and differential compositions of non-fucosylated/fucosylated xyloglucans, homogalacturonans, and highly branched rhamnogalacturonan-I polysaccharides, possibly contribute to longer elongation time and longer fiber phenotypes of *G. barbadense* relative to *G. hirsutum*. Our study also suggests that differential temporal compositions of arabinoxylans and glucuronoxylans might contribute to the variation in cellulose microfibril arrangement and the strength of fiber that exists between the two species of cotton. Comparative transcriptomic analysis identified differentially accumulated polysaccharide-synthesizing glycosyltransferases that may underlie differences in fiber quality between the two species. Transcripts encoding many cell wall-localized expansins were found to be more abundant in *G. barbadense* than in *G. hirsutum*, which could be a contributing factor for the longer fibers of *G. barbadense*. Overall, these findings expand our understanding of the molecular factors that contribute to fiber quality and provide insights for targeted cotton fiber improvement.

## 1 Introduction

Worldwide, cotton (*Gossypium* spp.) is the most important natural fiber used in the textile industry, and it is cultivated in over 80 countries. Among 50 naturally occurring cotton species, four have been domesticated, namely, two allotetraploids from Central and South America, *Gossypium hirsutum* (*Gh*, Upland or American cotton; AD1 genome), and *Gossypium barbadense* (*Gb*, Egyptian or Pima cotton; AD2 genome), respectively, and two African-Asiatic diploids, *Gossypium arboreum* (Tree cotton; diploid A2 genome) and *Gossypium herbaceum* (Levant cotton; diploid A1 genome) (Hu et al., 2021; Jareczek et al., 2023; Viot and Wendel, 2023). Essentially, in cotton, At (A-subgenome) and Dt (D-subgenome) represent the two ancestral genomes that merged during an ancient hybridization event, forming modern allotetraploid cotton species (*G. hirsutum* and *G. barbadense*), with At derived from A-genome diploids (like *G. arboreum/herbaceum*) and Dt from D-genome diploids (like *G. raimondii*), influencing traits like fiber quality and yield through differential gene expression and structural variation (Viot and Wendel, 2023). About 98% of the commercially cultivated cotton worldwide is from *Gh* and *Gb*. *Gh* offers higher yields and greater environmental adaptability, but only moderate fiber quality, suited for general-purpose textiles. In contrast, *Gb* cotton is grown only in selected environments, and its fiber is longer, stronger, thinner and finer (with less mass per unit length), which makes it preferred for spinning softer yarns used for luxury cotton clothing.

Cotton fibers are single-celled trichomes that elongate from the epidermis of the cotton seed coat, beginning near the day of anthesis. These seed trichomes (hereafter referred to as “cotton fibers” throughout this article) are among the longest plant cells, reaching 2.5 to 4 cm or more in length during 50 to 55 days post anthesis (DPA). Cotton fiber development is a highly coordinated, modular process consisting of at least five overlapping stages: 1) fiber cell initiation and tapering (–3 to 3 DPA); 2) elongation stage of primary cell wall (PCW) development (2 to 3 weeks); 3) transition period of PCW remodeling/early secondary cell wall (SCW) formation; 4) high cellulose accumulation/SCW thickening (3 to 5 weeks); and 5) maturation/drying (6 to 8 weeks) (Haigler et al., 2012; Kim, 2015; Huang et al., 2021; Yanagisawa et al., 2022). On average, longer fibers develop in *Gb*, perhaps because its elongation phase is longer in comparison to *Gh* (Hu et al., 2019).

The spinnable cotton fiber cell is primarily composed of dried cell wall (CW) polysaccharides. Cotton fiber development involves synchronized gene expression networks, hormone signaling and biosynthetic pathways, as well as physiological and developmental processes that drive dynamic changes in fiber CW polysaccharide composition (Jan et al., 2022; Jareczek et al., 2023; Swaminathan et al., 2024; Grover et al., 2025). The initial PCW is rich in pectic polysaccharides and xyloglucans (XGs) (Huwyler et al., 1979; Maltby et al., 1979; Singh et al., 2009), which confer plasticity to the CW, enabling rapid fiber elongation under high internal turgor pressure. As the fiber transitions to SCW production, pectins and XGs decrease in relative abundance and, in combination with the increase of cellulose content, enhance CW rigidity and strength (Tokumoto et al., 2002). After the transition stage, SCW is characterized by massive cellulose accumulation in fibers, followed by maturation, programmed cell death, lumen collapse, and fiber dehydration. The matured, dried fibers used for yarn and textile manufacturing contain more than 95% cellulose with traces of non-cellulosic polysaccharides, glycoproteins, sugars, and minerals (Haigler et al., 2012).

The availability of superior quality *Gb* (long, strong and finer) and moderate quality *Gh* accessions and temporally sampled polysaccharides during fiber PCW and SCW synthesis makes the cotton fiber an excellent model to gain insight into the molecular and biochemical processes responsible for cotton fiber development (Lacape et al., 2012; Li et al., 2013; Tuttle et al., 2015; Hu et al., 2019; Zhang et al., 2022; Liu et al., 2023). This understanding, in turn, will inform efforts to improve fiber quality traits in both allopolyploid cotton species.

Comparative “glycomic” studies on fiber CW polysaccharides across different cotton species established that dynamic changes occur in the non-cellulosic polysaccharide epitopes of pectins/XGs/xylans, in addition to cellulose, during fiber elongation, transition, and early SCW thickening stages (Singh et al., 2009; Avci et al., 2013; Hernandez-Gomez et al., 2015, 2017; Guo et al., 2019; Pettolino et al., 2022; Swaminathan et al., 2024). These studies showed that fiber length in various species is determined during PCW biosynthesis/remodeling in the elongation stage, while fiber strength is influenced during SCW biosynthesis/thickening stage. In addition to CW polysaccharide composition and the extended fiber elongation stage, subtle variations in the timing of cellulose deposition in fiber CW is an essential factor for quality differences between *Gb* and *Gh* fibers (Avci et al., 2013; Li et al., 2013; Rajasundaram et al., 2014).

Earlier, we carried out glycome, transcriptome, and proteome profiling on *Gh* (accession TM-1) and identified critical polysaccharide epitopes and potential glycosyltransferases that synthesize them (Swaminathan et al., 2024; Grover et al., 2025; Lee et al., 2025a). In the present study, we analyzed *Gb* (accession 3-79) using precisely the same approaches and compared the two cotton species (*Gh* and *Gb*) with respect to glycome, transcriptome, and proteome profiling using temporally dense sampling, in parallel with our earlier study (Swaminathan et al., 2024). Thus, we compared developing fibers of *Gh* (accession TM-1) and *Gb* (accession 3-79) daily from 6 to 25 DPA. These 20 consecutive days of sampling covered critical phases of fiber development, including PCW synthesis, transitions from PCW to SCW synthesis, and SCW synthesis, during which gene expression varies between *Gh* and *Gb* (Al-Ghazi et al., 2009; Liu et al., 2023). A broad collection of polysaccharide-specific monoclonal antibodies was utilized for glycome profiling. The primary focus of our study was to elucidate the differences in the dynamics of polysaccharide epitope arrangement during fiber development between the two species and evaluate their potential contribution to fiber quality. In order to study these differences a comparative analysis was conducted between the polysaccharide epitopes and glycosyltransferase genes (from transcriptomic data) involved in producing enzymes that synthesize the polysaccharide epitopes. Although the CW polysaccharide epitope content during different stages of fiber development is changed by the action of both the glycosyltransferases and CW glycosyl hydrolases, in this study, we focused exclusively on the glycosyltransferases. Analysis of CW glycosyl hydrolases will be carried out in the future in a separate study. In addition, we compared the transcriptome of expansins, the CW-loosening proteins, which were reported as one of the most important factors that contributes to fiber elongation (Sampedro and Cosgrove, 2005; Xu et al., 2013; Li et al., 2016; Lv et al., 2020). Our large-scale, high-temporal-resolution comparative multi-omics analyses between two cotton species revealed many dynamically remodeled polysaccharides and the associated differentially accumulated glycosyltransferases and expansins, which may contribute to the distinct fiber quality traits in the two species. These findings provide valuable insights that could result in new cotton breeding strategies for improved fiber traits.

## 2 Materials and methods

### 2.1 Cotton plant growing conditions and boll collection

Two different cotton species, *Gh* (accession TM-1) and *Gb* (accession 3-79), were grown synchronously under controlled conditions in a growth chamber (Conviron E-15, Controlled Environments Inc. N.D., USA) as previously described (Grover et al., 2025; Lee et al., 2025a, 2025b). Plants were grown individually in a two-gallon pot containing a potting mix (4:2:2:1 ratio of soil:perlite:bark:chicken grit). Growth chambers were set for 16 h days at 28°C and 500 µmol m^-^² s^-^¹ of light. Flowers were self-pollinated before noon every day and tagged. For 20 different time points (from 6 to 25 DPA), bolls were collected at mid-day and stored at -80° C. At each time point, three biological replicate bolls were used for glycome, proteome and transcriptome analysis.

### 2.2 CW and polysaccharide extraction

From the cotton fiber, the CW and the buffer-soluble and alkali-soluble polysaccharide fractionswere extracted as per the established protocol (Avci et al.,2013; Swaminathan et al., 2024). In brief, using a razor blade and tweezers, fibers were isolated from the developing seeds, and care was taken to avoid the seed coat while harvesting the fibers. Harvested cotton fibers from a single boll were ground into powder using liquid nitrogen, and the CW was then extracted from the powdered fiber using solvents (Swaminathan et al., 2024). Polysaccharide fractions were sequentially extracted from the CW by first using a 50 mM CDTA:50 mM ammonium oxalate (1:1) buffer, followed by 4 M KOH to extract buffer-soluble and alkali-soluble polysaccharides, respectively (Avci et al., 2013; Swaminathan et al., 2024). The buffer-soluble and alkali-soluble extracts were adjusted to pH 7.0, and dialyzed (SnakeSkin Dialysis Tubing, MWCO: 3.5 kDa; ThermoFisher Scientific) for 4 days using only sterile double-distilled water to remove the salts (Avci et al., 2013). The dialyzed extracts were frozen, lyophilized to dryness, and weighed. The final pellet, which contained a mixture of amorphous and crystalline celluloses, was weighed. The crystalline cellulose content present in the final pellet was measured by using the Updegraff reagent (acetic acid: nitric acid: water, 8:1:2 v/v) (Updegraff, 1969) (**Supplementary Data Sheet 1**).

### 2.3 Glycome profiling of epitopes of buffer-soluble and alkali-soluble polysaccharide fractions

Glycome profiling was carried out using a standard protocol (Pattathil et al., 2010; Pattathil et al.,2012). Initially, the sugar content in each of the buffer-soluble and alkali-soluble polysaccharide fractions from each sample was estimated by the phenol-sulfuric acid method (Pattathil et al., 2012). Later, the polysaccharide sample was dissolved in water. An equal amount of 3 µg (50 µl/well from a 60 µg/µl solution) was added to each well in 96-well Enzyme-Linked Immunosorbent Assay (ELISA) plates (Costar 3595). The polysaccharides were allowed to bind to the bottom of the well by drying in an oven at 37°C. Glycome profiling was conducted using an ELISA method as described earlier (Swaminathan et al., 2024) with 71 polysaccharide epitope-specific antibodies selected based on the literature (Avci et al., 2013; Thorne et al., 2023). In brief, the ELISA procedure includes the following steps: the polysaccharide coated wells were blocked with nonfat dry milk solution, incubated for 1 h, washed once, incubated with glycome antibodies for 1 h, washed three times, incubated with peroxide-conjugated secondary antibody for 1 h, washed five times, incubated with TMB-peroxidase substrate solution for 40 min, the reaction was stopped by adding 1.0 N sulfuric acid and the color development was detected at 450 nm wavelength in an ELISA plate reader and the optical density (OD) reading from each sample were used for further analysis. These selected 71 antibodies detect the backbone and decorated side chains of the pectins, homogalacturonan (HG) and rhamnogalacturonan-I (RG-I), as well as the hemicelluloses, XGs and xylans (Xyls), and callose. The specific details of each antibody used in this study, along with the polysaccharide epitopes detected, are reported in the supplementary information (**Supplementary Data Sheet 2**; **Supplementary Figure 1**). Data from the ELISA for each epitope were obtained from either buffer-soluble oralkali-soluble polysaccharide fractions of the same sample (**Supplementary Data Sheet 3**). To distinguish between the two types of polysaccharide fractions, for the naming of epitopes, we used a naming convention where a suffix of “-C” refers to the buffer-soluble fraction (50 mM CDTA:50 mM ammonium oxalate buffer extracts) and “-K” refers to the alkali-soluble fraction (4 M KOH extracts).

### 2.4 Self-organizing map clustering

The SOM is a robust unsupervised machine-learning method that has built-in Cosine similarity(statistical distance metrics) used for clustering analysis/pattern discovery (Kohonen, 1990). An SOM algorithm, based on an inner product distance metric, was applied to the ELISA-based glycome profiling results (as described in previously established protocols; Lee et al., 2025a; Yang et al., 2025) to cluster the abundance profiles of glycome epitopes from two separate interpolated datasets: buffer-soluble and alkali-soluble polysaccharide fractions from two different species of cotton (Wehrens and Kruisselbrink, 2018). We optimized the number of clusters by balancing between over−segmentation (leading to empty or single-member clusters) and under−segmentation (losing biologically meaningful groups), ultimately selecting a 12-cluster SOM. Thus, SOM grid structures consisted of 3 rows and 4 columns for both buffer-soluble and alkali-soluble polysaccharide fractions datasets. The resulting clusters represent groups of glycome epitopes with similar abundance trajectories. In the SOM analysis, the data from both species for each of the fractions were combined, and two different colors were used to maintain the identity of polysaccharide epitopes. The SOM was performed in R Studio (Version 2024.12.0.467, R Studio; R Core Team, 2022) using the supersom function in the kohonen package (Version 3.0.12, Wehrens and Kruisselbrink, 2018). The p-value threshold determining significance was 0.05. Based on the significant differences, the polysaccharide epitopes were grouped into mainly three different categories (presented in figures and supplementary information).

### 2.5 RNA extraction and RNA sequencing

In parallel to glycome profiling, RNAseq was carried out for the cotton fibers collected simultaneously for the same days (6 to 25 DPA) (Grover et al., 2025). Briefly, a modified version of the Sigma Plant Spectrum Total RNA kit (Sigma-Aldrich) was used to extract RNA from the fibers. Library construction (NEBNext Ultra II RNA Library Prep Kit) and sequencing (as PE150; Illumina NovaSeq 6000) were conducted by the Iowa State University DNA facility. Data were processed using Trimmomatic version 0.39 (trimmomatic/0.39-da5npsr) (Bolger et al., 2014) from Spack (Gamblin et al., 2015) for read and quality trimming. The transcripts of each gene were annotated based on the published reference *Gh* genome (Chen et al., 2020) and *Gb* genome (Chen et al., 2020). The transcripts per million (TPM) output by Kallisto for each sample were imported into R/4.2.2 (R Core Team, 2022), and RNA-seq quality was assessed by consistency of the number of genes expressed over time and among replicates. The number of expressed genes per sample (TPM > 0) was plotted across developmental time using ggplot2, and visual outliers were discarded. RNAseq reads of cotton fibers collected from 6 to 25 DPA were deposited into the NCBI-SRA under PRJNA1099209 (*G. hirsutum*) and PRJNA1222456 (*G. barbadense*). Differential gene expression analysis was performed using DESeq2, and p-values were adjusted using the False Discovery Rate (FDR) Benjamini-Hochberg method (Benjamini and Hochberg, 1995). Differential gene expression was determined, where p-values were less than 0.05.

### 2.6 Transcriptomic analysis of glycosyltransferase genes

Glycosyltransferases and enzymes that decorate polysaccharides play key roles in the synthesis offiber CW polysaccharides. To compare the transcript levels of these enzymes between the two species,we first compiled a list of known or putative *Arabidopsis thaliana* glycosyltransferase genes based on our previous study and other studies (Swaminathan et al., 2024). Subsequently, the gene sequences of *Arabidopsis* proteins were used to identify the homologous genes of *Gh* and *Gb* from the Phytozome 13 (https://phytozome-next.jgi.doe.gov) and the Cotton Functional Genomics Database (https://cottonfgd.net) (**Supplementary Data Sheet 4**). Based on this information, transcript profiles of predicted glycosyltransferases and other polysaccharide-decorating enzymes in both *Gh* and *Gb* were generated from the transcriptome data (Grover et al., 2025). Comparisons of profiles of transcripts of these enzymes between *Gh* and *Gb* and their sub-genomes (At and Dt) were conducted.

### 2.7 Microsomal protein profiling

Since the glycosyltransferases that synthesize polysaccharides are membrane proteins, themicrosomal protein fraction (P200) was isolated from intact *Gb* fiber from 6 to 25DPA, as described previously (Lee et al., 2025a). Briefly, apoplastic proteins and extracellular vesicles were removed from the intact ovules by dipping each intact ovule into 5 mL of pre-chilled microsome isolation buffer (MIB) for 10 min. Subsequently, the fiber tissues were isolated, homogenized in cold MIB using a Polytron homogenizer, and filtered through cheesecloth. From the filtrate, microsomes were enriched by ultracentrifugation (200,000 x g for 20 min at 4°C). The final pellet was mixed with 8 M Urea to denature membrane proteins, digested with trypsin, and desalted using C18 columns (Lee et al., 2025a, 2025b). The peptides were analyzed using Bruker’s TIMS-TOF HT (Bruker Daltonics GmbH) mass spectrometer (MS), which was coupled with a reverse-phase liquid chromatography system, nanoElute2 (Bruker Daltonics GmbH). The MS raw data were processed using the directDIA™ approach in Spectronaut software (Version 19.4, Biognosys) according to the vendor’s recommendations. The spectral library was constructed using a cotton FASTA file containing 55,237 entries and DPA-pooled DDA runs. Cysteine Carbamidomethylation was set as a fixed modification, while protein N-terminal acetylation and methionine oxidation were set as variable modifications. Trypsin was specified as the digestion enzyme, allowing a maximum of two missed cleavages. The FDR was set to 1% at both the peptide and protein levels. Quantification was based on MS2-level fragment ion extracted ion chromatograms (XICs), with protein abundances calculated using Spectronaut’s MaxLFQ algorithm. The glycosyl transferase proteomic data of *Gb* fibers presented in this study (**Supplementary Data Sheet 5**) is part of a larger whole proteome dataset. The whole *Gb* fiber proteomedataset consisting of 13,357 proteins including glycosyl transferases is presented in **Supplementary Data Sheet 5**.

### 2.8 Statistical analysis

The experimental data, including Pearson correlation coefficient (PCC) analysis, were subjected to statistical analysis using R software (Version 2024.12.0.467, R Studio; R Core Team, 2022). PCC value with >0.7 was considered the best correlation profile pattern. Statistical significance was set at p < 0.05, and p-values were adjusted using the FDR. The t-test was carried out to compare the polysaccharide content at different DPA to find out the statistical significance in R Studio (Version 2024.12.0.467, R Studio; R Core Team, 2022). Exact p-values are presented in the **Supplementary Table**, and p-values were adjusted using the FDR. The p-value threshold determining significance was 0.05.

## 3 Results

### 3.1 Polysaccharide composition of *G. hirsutum* and *G. barbadense* cotton fibers during development (6–25 DPA)

**Figure 1:**
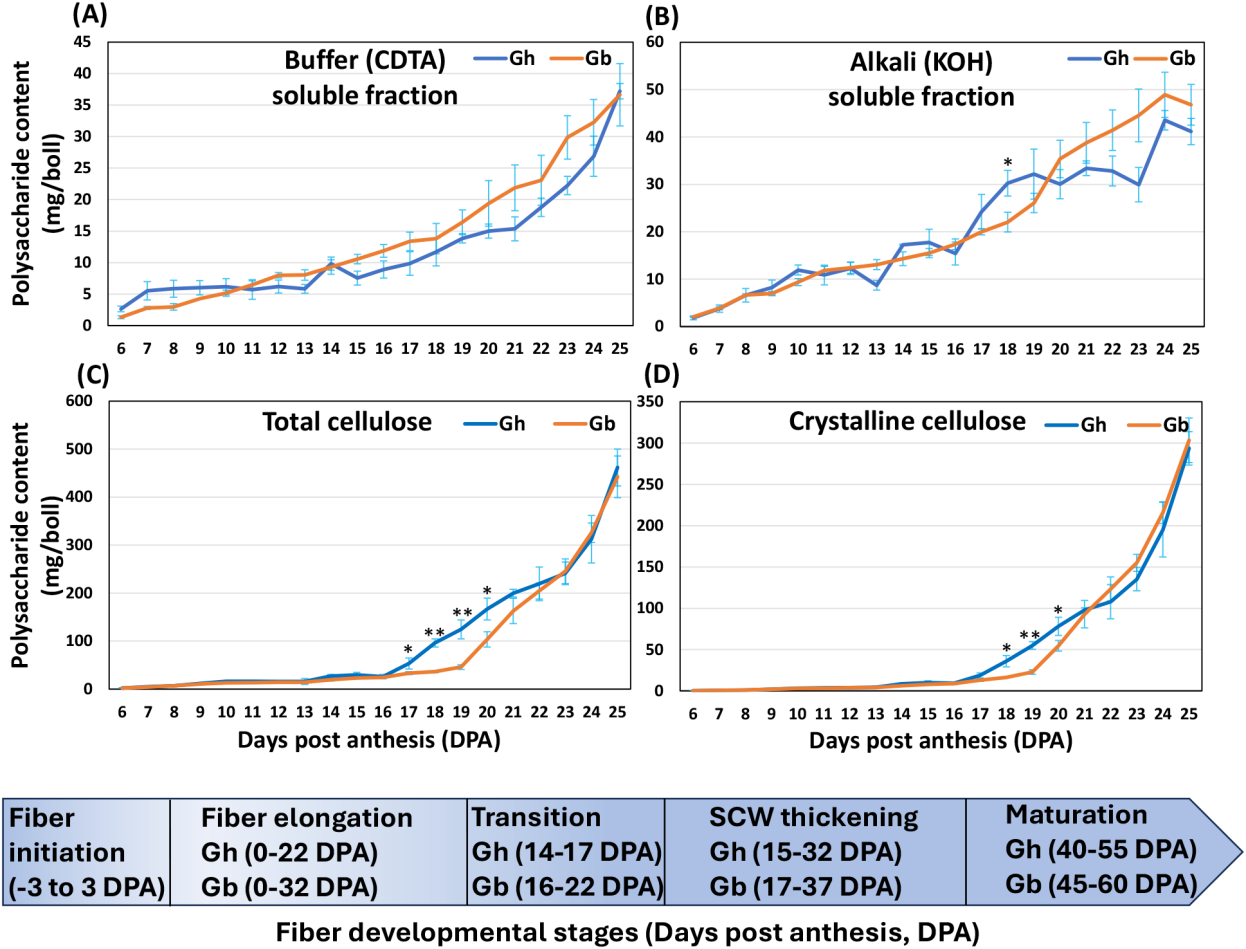
Polysaccharide contents of fiber CW fractions in *Gh* and *Gb* during development (6–25 DPA). **(A)** Buffer-soluble (50 mM CDTA-50 mM Ammonium oxalate extract) polysaccharide content of *Gh* and *Gb* fibers. **(B)** Alkali-soluble (4 M KOH extract) polysaccharide content of *Gh* and *Gb* fibers. **(C)** Total cellulose content of *Gh* and *Gb* fibers. **(D)** Crystalline cellulose content of *Gh* and *Gb* fibers. Data represents averages ± SE of three biological replicates. Significant differences are shown as *P < 0.05 and **P < 0.01. The panel at the bottom of the figure shows five different fiber developmental stages and approximate time period for each stage.

The buffer-soluble fractions (50 mM CDTA:50 mM ammonium oxalate buffer extracts), alkali-soluble fractions (4 M KOH extracts), and cellulose were extracted from cotton fiber CW (6 to 25 DPA), lyophilized, weighed and averaged (**Figure 1**; **Supplementary Data Sheet 1**). As expected, the amount of cellulose and non-cellulosic polysaccharide fractions per boll increased gradually from 6 to 25 DPA in both species.

At 6 DPA, total cellulose content (consisting of both amorphous and crystalline cellulose) was 2.3 mg/boll and 2.1 mg/boll, for *Gh* and *Gb*, respectively (**Figure 1**; **Supplementary Data Sheet 1**). Cellulose content gradually increased in both species after this time; however, the rate of increase was slower in *Gb*. By 16 DPA, cellulose content reached 25.9 mg in *Gh* and 24.3 mg in *Gb*. After 16 DPA, the cellulose content of *Gh* started to increase rapidly (53.3 mg at 17 DPA; 124.2 mg at 19 DPA), coinciding with the SCW thickening stage (Li et al., 2013), and reached 461.6 mg at the end of 25 DPA. Interestingly, unlike *Gh*, the cellulose content of *Gb* increased gradually and slowly after 16 DPA and reached only about 45.9 mg at the end of 19 DPA, and after that, it started to increase rapidly (103.6 mg at 20 DPA; 162.6 mg at 21 DPA), coinciding with the SCW thickening stage, reaching 442.3 mg at the end of 25 DPA, a comparable level to that of *Gh*. The amount of estimated crystalline cellulose content of both species showed profile patterns similar to that of total cellulose content. Crystalline cellulose content was about 0.2 mg per boll at 6 DPA and about 9.0 mg at 16 DPA in both species (**Figure 1**; **Supplementary Data Sheet 1**). From 17 DPA onwards, crystalline cellulose content increased rapidly in *Gh*, but more slowly in *Gb*. At 20 DPA, crystalline cellulose content was 78.2 mg in *Gh* and 54.7 mg in *Gb*. After 20 DPA, this amount increased rapidly (at 21 DPA, 97.8 mg in *Gh* and 92.8 mg in *Gb*). At 25 DPA, the crystalline cellulose content was estimated to be 293.6 mg in *Gh* and 303.5 mg in *Gb*. Overall, in *Gb* there was a three-day delay in cellulose content accumulation relative to *Gh*.

The content of buffer-soluble polysaccharides at 6 DPA was lower in *Gb* (1.3 mg) in comparison with *Gh* (2.9 mg). The relative content of the buffer-soluble polysaccharide fraction gradually increased after 6 DPA, and the increment was faster after 17 DPA for *Gh* and 20 DPA for *Gb*, which coincided with their respective increase in cellulose content. The relative amount of buffer-soluble polysaccharide fractions reached 37.2 mg in *Gh* and 36.6 mg in *Gb* at the end of 25 DPA. The content of alkali-soluble polysaccharides was 2.1 mg for *Gh* and 2.0 mg for *Gb* at 6 DPA and 15.4 mg and 17.4 mg, respectively, at the end of 16 DPA. Similar to the buffer-soluble polysaccharide fraction, the relative content of alkali-soluble polysaccharides started to increase at a faster rate after 16 DPA, coinciding with an increase in the cellulose content, and reached 41.2 mg and 46.8 mg in *Gh* and *Gb*, respectively, at the end of 25 DPA.

### 3.2 Glycome, transcriptome, and proteome analyses of *Gb* fiber

We conducted comprehensive glycome, transcriptome, and proteome profiling of only *Gb* fibers (collected daily from 6 to 25 DPA). As described in our previous study (Swaminathan et al., 2024), glycome profiling of cotton fiber CW polysaccharide epitopes was performed using 71 monoclonal antibodies targeting diverse CW polysaccharide epitopes (**Supplementary Figure 1**; **Supplementary Data Sheet 2**), and the glycome profiling data of *Gb* fiber are presented in **Supplementary Data Sheet 3**. Then we curated a list of candidate glycosyltransferases (**Supplementary Data Sheet 4**). Glycosyltransferases are membrane proteins isolated in the microsomes during proteinpurification. So, in order to identify the glycosyltransferases responsible for synthesizing thepolysaccharides corresponding to the observed epitope patterns (**Supplementary Data Sheet 3**), we integrated transcriptome and proteome data from the microsomal (P200) fraction isolatedat each developmental stage (6 to 25 DPA) (**Supplementary Data Sheet 5**). PCC analysis was used to integrate glycome profiles with gene and protein accumulation data.

**Figure 2:**
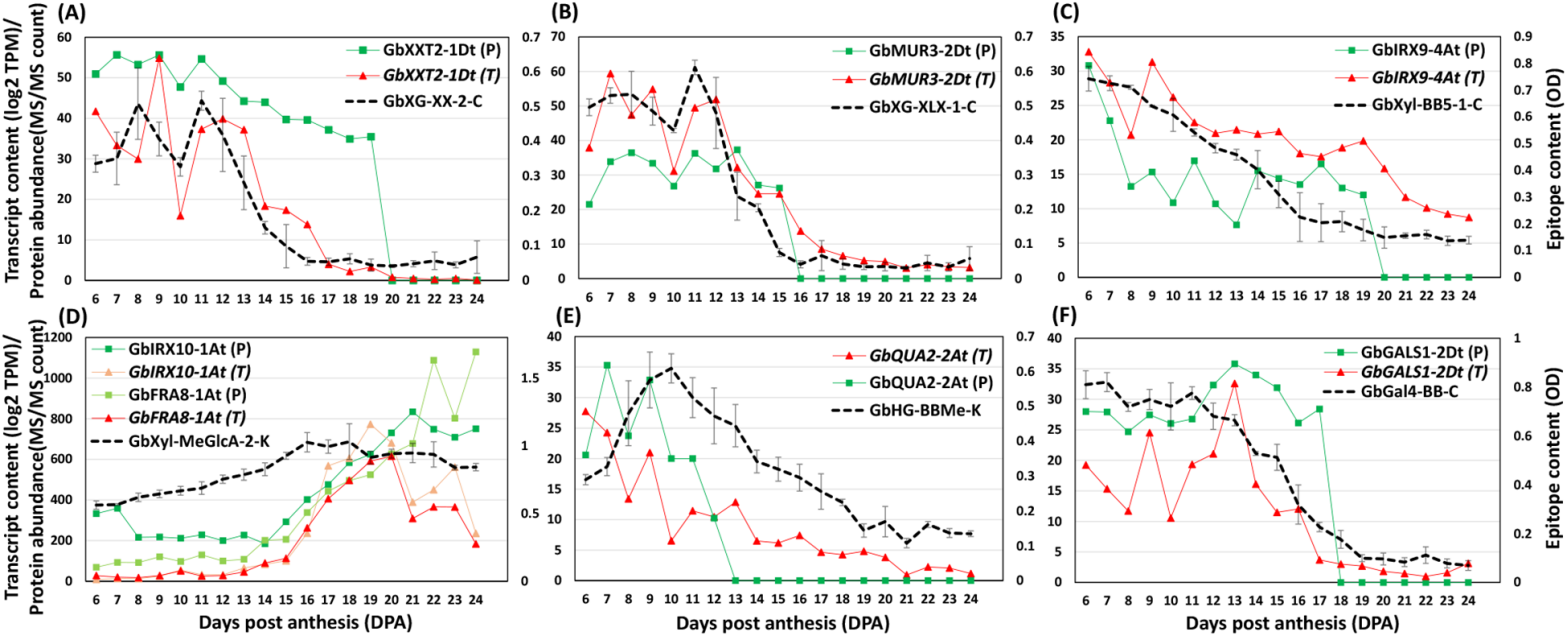
Representative profiles of *Gb* polysaccharide epitopes and significantly correlated transcripts and protein profiles of glycosyltransferases involved in synthesizing these epitopes. Polysaccharide epitopes are represented by a black dotted line, transcripts (T) by a red solid line, and proteins (P) by a green solid line. **(A)** Profiles of a xylosylated glucan epitope (GbXG-XX-2-C) and correlated representative transcript and protein of a XG xylosyltransferase (GbXXT2-1Dt). **(B)** Profiles of a galactosylated XG epitope (GbXG-XLX-1-C) and correlated representative transcript and protein of a XG galactosyltransferase (GbMUR3-2Dt). **(C)** Profiles of a Xyl backbone epitope (GbXyl-BB5-1-C) and correlated representative transcripts and proteins of a xylosyltransferase (GbIRX9-4At). **(D)** Profiles of a methylated-glucuronoxylan epitope (GbXyl-MeGlcA-2-K) and correlated representative transcripts and proteins of a xylosyltransferase (GbIRX10-1At) and a transferase involved in reducing end synthesis (GbFRA8-1At). **(E)** Profiles of a methylated-HG epitope (GbHG-BBMe-K) and correlated representative transcript and protein of a methyltransferase (GbQUA2-2At). **(F)** Profiles of a β-1,4-galactan secondary backbone epitope of RG-I (GbGAL4-BB-C) and correlated representative transcript and protein of a β-1,4-galactan synthase (GbGALS1-2Dt). Protein quantification values represent Log2-transformed MaxLFQ intensities derived from MS2 fragment ion areas (XIC).

In this study, we focused on polysaccharide-synthesizing glycosyltransferases and associatedpolysaccharide-decorating enzymes, such as methyltransferases and acetyltransferases, that areresponsible for the biosynthesis and modification of CW polysaccharides analyzed in the glycome profiles. Among the 13,357 proteins detected in the P200 proteome of *Gb* fibers, we identified only 93 glycosyltransferases (**Supplementary Data Sheet 5**) that belong to the 550 putative glycosyltransferases identified by homology from*Arabidopsis* (**Supplementary Data Sheet 4**). About 38% of mRNA/protein pairs in P200 had PCC values > 0.50 (~15% > 0.7 PCC),suggesting that their protein abundance could be explained by transcriptional control to some extent(**Supplementary Data Sheet 5**). Also, the PCC analysis revealed significant correlations between a few of thepolysaccharide epitopes and the profile patterns of corresponding glycosyltransferases at bothtranscript and protein levels (**Supplementary Data Sheet 6**), with representative correlated profiles shown in **Figure 2**. Overall, from PCC analysis, higher correlations were observed between XGs and theirassociated glycosyl transferases, followed by HGs and Xyls (**Supplementary Data Sheets 5**, **6**). A minimal correlation was observed between RG-I and callose, as well as between RG-I and the associated glycosyltransferases.

For XG biosynthesis or modification, four XG xylosyltransferases (XXTs) (GbXXT2-1Dt, GbXXT2-2At, GbXXT3-1At, and GbXXT3-2At/Dt) were detected in the proteome. These XXTs exhibited protein accumulation profiles that closely mirrored the abundance patterns of xylosylated XG epitopes (GbXG-XX and GbXG-XXXG), with strong correlations (PCC ≥ 0.7) and with profiles gradually decreasing from 6 to 25 DPA of fiber development (**Figure 2**; **Supplementary Data Sheet 6**). Notably, two out of the four *XXT*s (*GbXXT2-1Dt* and *GbXXT3-1At*) also displayed similar transcript profiles, with PCC values greater than 0.75. Similarly, the transcript levels of two of four XG galactosyltransferases (*GbMUR3-2At* and *GbMUR3-2Dt*) showed high correlations with PCC values of 0.82 and 0.92, respectively. Both MURs also exhibited strong correlations (PCC = 0.7 - 0.95) with galactosylated XG epitopes (GbXG-XLX and GbXG-L), as shown in **Figure 2** and detailed in **Supplementary Data Sheet 6**.

The analysis revealed that many of Xyl-related glycosyltransferases, identified from both the proteome and transcriptome datasets, showed significant correlations with the profiles of xylan backbone (Xyl-BB) and branched Xyl epitopes (GbXyl-GlcA and GbXyl-MeGlcA) (**Figures 2C, 2**; **Supplementary Data Sheet 6**). Notably, glycosyltransferases such as GbIRX9-3At/Dt, GbIRX9-4At, GbIRX10-1At/Dt,GbIRX15-2At/Dt, GbFRA8-1At/Dt, GbGUX1-1At/Dt, GbGUX2-2Dt, and GbGXMT3-1At/Dt exhibited strongcorrelation (PCC > 0.7) with GbXyl-MeGlcA epitopes (**Supplementary Data Sheet 6**). Among them, xylosyltransferase GbIRX9-3At/Dt also showed a strong correlation with the corresponding transcript profiles (PCC = 0.8) (**Figure 2**; **Supplementary Data Sheet 6**). Also, a few methyltransferases (GbRWA1-1At/Dt, GbRWA1-4At/Dt) and two acetyltransferases(GbTBL32-1Dt, GbTBL32-2Dt) correlated with GbXyl-MeGlcA epitopes (**Supplementary Data Sheet 6**). For HG biosynthesis, GbQUA2-2At/Dt and GbTBR-5At/Dt showed strong correlations between their transcript and protein accumulation profiles (PCC > 0.7), and their protein levels were highly correlated with the abundance patterns of homogalacturonan (GbHG) epitopes (PCC > 0.85) (**Figure 2**; **Supplementary Data Sheet 6**). For RG-I biosynthesis, the profile of GbGal4-BB epitopes showed a strong correlation with the protein accumulation profile of GbGALS1-2Dt (PCC = 0.95) (**Figure 2**; **Supplementary Data Sheet 6**).

### 3.3 Glycome profiling: heat maps, and SOM of diverse epitope patterns of polysaccharides in *Gh* and *Gb* fibers

To find out the differences in polysaccharide epitope compositions and differences in temporal dynamics of polysaccharides between *Gh* and *Gb* fibers, ELISA absorbance data from glycome profiling of *Gh* and *Gb* fibers at 6–25 DPA (**Supplementary Figure 1**; **Supplementary Data Sheet 3**) were visualized as heat maps and grouped into SOM clusters. ELISA data for each epitopewere obtained from two different fractions of the same sample: the buffer-soluble and alkali-solublefractions (**Supplementary Data Sheet 3**). ELISA data from *Gh* and *Gb* were analyzed together, but the identities of all 71 epitopes were maintained separately for both species.

**Figure 3:**
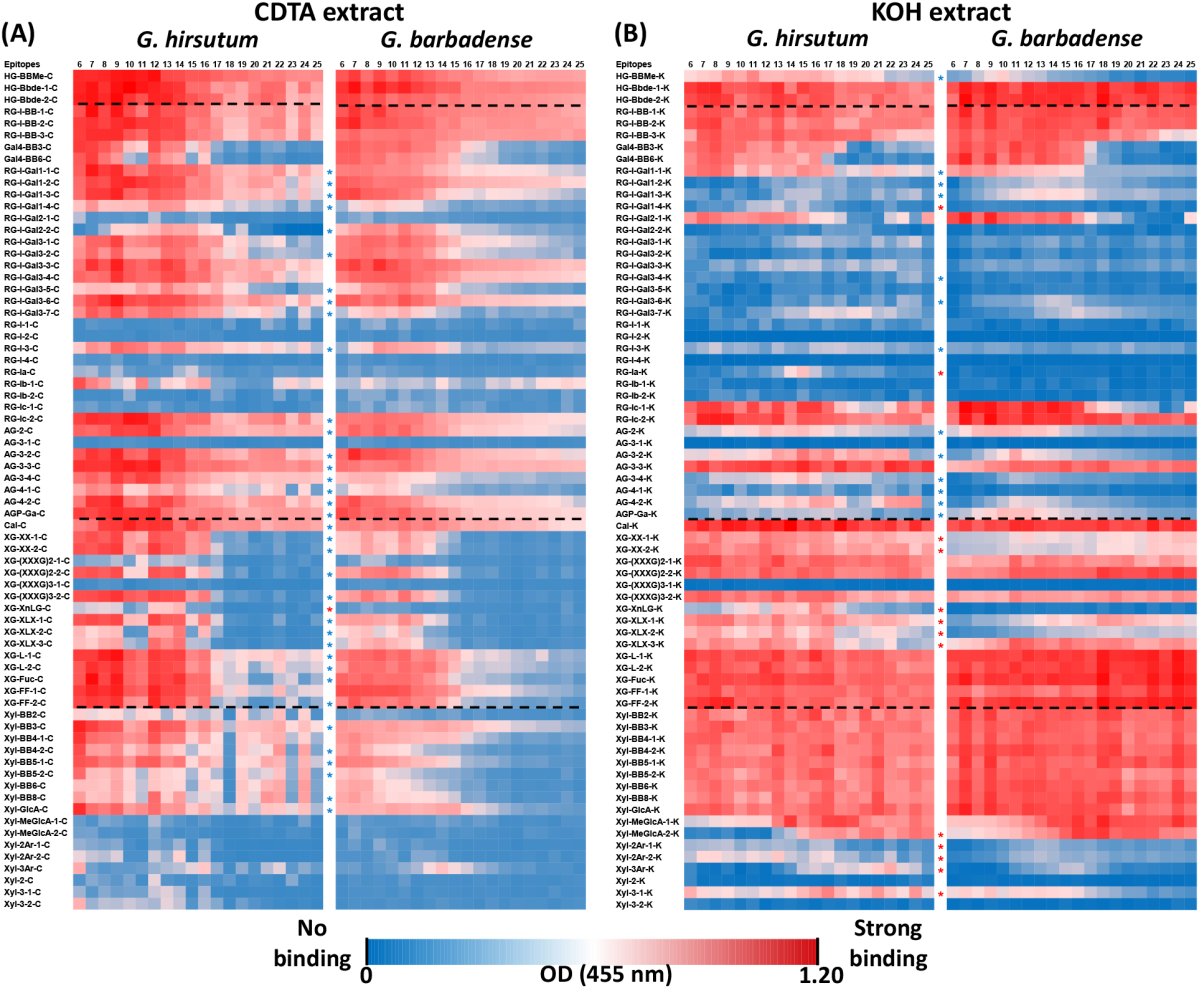
Heat maps of glycome profiled 71 different polysaccharides epitopes in fiber tissue of two cottonspecies during development (6–25 DPA). **(A)** Heat maps of glycome profiledepitopes from the buffer-soluble (50 mM CDTA-50 mM Ammonium oxalate extract) polysaccharides fractions of *Gh* and *Gb*. Data represent averaged optical density (OD) values from three biological replicates. **(B)** Heat maps of glycome profiled epitopes from the alkali-soluble (4 M KOH extract) polysaccharide fractions of *Gh* and *Gb*. Data represent averaged OD from three biological replicates. Epitopes from either buffer-soluble (50 mM CDTA-50 mM Ammonium oxalate extract) or alkali-soluble (4 M KOH extract) fractions are denoted by the suffix “-C” and “-K”, respectively. Epitopes exhibiting similar patterns, but varying in content between the two species are marked with blue stars, while epitopes with different patterns between the species are marked with red stars. Detailed glycan epitope binding specificities of the 71 monoclonal antibodies used in this study are provided in **Supplementary Data Sheet 2**.

Heat maps were generated separately for buffer-soluble polysaccharide and alkali-soluble polysaccharide fractions from both species (**Figure 3**). In these heat maps, epitopes were matched one-to-one between *Gh* and *Gb* to highlight their differential distribution during fiber development (**Figure 3**). The heat maps of buffer-soluble polysaccharides showed that many of the highly branched RG-I, arabinogalactan (AG), XG, and some Xyl epitopes were present in lower amounts in *Gb*. Also, their quantity dropped at earlier stages of fiber development in *Gb* in comparison with *Gh* (**Figure 3A**). The heat maps of alkali-soluble fractions showed that many of the XG and Xyl epitopes were present at high levels and in comparable amounts between both species. In addition, the heat maps clearly showed that the profile patterns and content of some of the XG (XG-XX-1-K, XG-XX-2-K, XG-XLX-1-K, XG-XLX-2-K, XG-XLX-3-K, XG-XnLG-K), Xyl (Xyl-2Ar-1-K, Xyl-2Ar-2-K, Xyl-3Ar, Xyl-MeGlcA-2-K, Xyl-3-1-K), and of the RG-I (RG-I-1-4-K, RG-Ia-K) epitopes were prominently different between the two species (**Figure 3B**).

**Figure 4:**
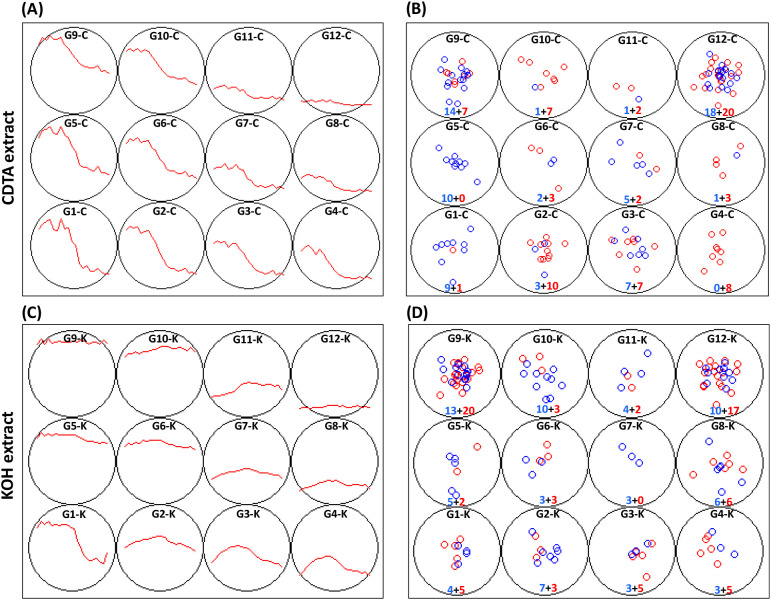
SOM of glycome profiled polysaccharide epitope patterns in cotton fibers from two species duringdevelopment (6–25 DPA). **(A)** SOM groups (G1-C to G12-C) representing the glycomeepitope profiles of buffer-soluble polysaccharides (50 mM CDTA-50 mM Ammonium oxalate extract, suffix “-C”). **(B)** Distribution of epitopes from *Gh* (blue circles) and *Gb* (red circles) in each SOM group shown in **(A)**. **(C)** SOM groups (G1-K to G12-K) representing the glycome epitope profiles of alkali-soluble polysaccharide (4 M KOH extract, suffix “-K”). **(D)** Distribution of epitopes from *Gh* (blue circles) and *Gb* (red circles) in each SOM group shown in **(C)**. Profiles in **(A, C)** represent averaged data from three biological replicates. Each of the 12 profiles shown in **(A, C)** starts from 6 DPA (left side) and ends with 25 DPA (right side). In **(B, D)**, each colored circle represents one specific glycome profiled epitope from either of the cotton species, and the numbers indicate the total number of epitopes comes under specific profile shown in **(A, C)**, respectively. The specific details of epitopes comes under each profile is listed in **Supplementary Data Sheet 7**.

Next, the glycome profiles from *Gh* and *Gb* were combined and clustered using an unbiased SOM approach to investigate the dynamics of epitope compositions across fiber development. In total, 12 SOM cluster groups of glycome profile epitope patterns were generated. SOM analysis was mainly carried out to identify similarities and differences of polysaccharide epitope profiles between the two species (cluster G1-C to G12-C for buffer-soluble polysaccharide fractions and cluster G1-K to G12-K for alkali-soluble polysaccharide fractions) (**Figure 4**; **Supplementary Data Sheets 7**, **8**). Further, the SOM grouping analysis helped us easily categorize epitopes at a finer levelbased on the profile patterns and the number of specific epitopes, as well as at the species level.Members present in each SOM cluster are provided in **Supplementary Data Sheet 7**. The SOM profiles for buffer-soluble polysaccharides showed that most polysaccharide epitopes had higher contents at 6 DPA, then gradually or rapidly decreased to lower levels at 25 DPA (**Figure 4A**). However, the total amount of buffer-extractable components continued to increase during fiber development (**Figure 1A**). This indicates that some polysaccharide epitopes remained undetected in buffer-soluble fractions of fiber CW by the antibodies used in our study. Epitopes in clusters G8-C, G11-C, and G12-C remained low throughout all DPAs. SOM clustering of alkali-soluble polysaccharides showed greater variability in epitope patterns, with some increasing or decreasing gradually or rapidly, while others peaked between 12 and 16 DPA during fiber development (**Figure 4C**).

**Figure 5:**
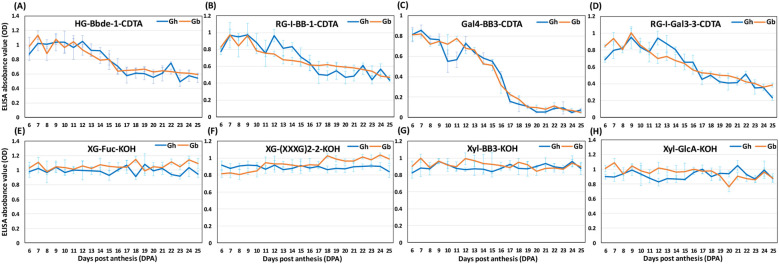
Glycome profiles of polysaccharide epitopes with equal content and same pattern between*Gh* and *Gb* fiber across development (6–25 DPA).**(A–H)** Representative profiles of polysaccharide epitopes listed in **Supplementary Data Sheet 8A** are shown here. The profiles of *Gh* (blue) and *Gb* (orange) show averaged optical density (OD) values of three biological replicates with standard error bars. Epitopes from either buffer-soluble (50 mM CDTA-50 mM Ammonium oxalate extract) or alkali-soluble (4 M KOH extract) polysaccharide fractions are denoted by the suffix “-CDTA” and “-KOH”, respectively.

**Figure 6:**
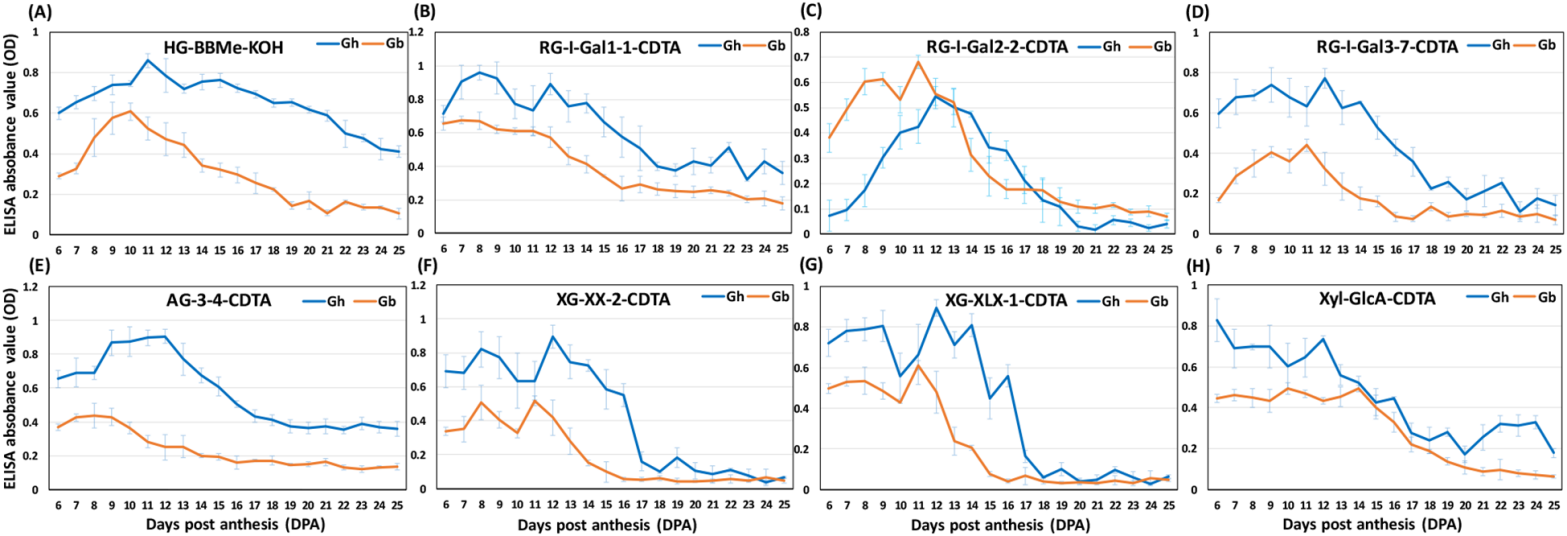
Glycome profiles of polysaccharide epitopes with the same pattern, but varying content between*Gh* and *Gb* fiber across development (6–25 DPA).**(A–H)** Representative profiles of polysaccharide epitopes listed in **Supplementary Data Sheet 8B** are shown here. The profiles of *Gh* (blue) and *Gb* (orange) show averaged optical density (OD) values of three biological replicates with standard error bars. Epitopes from either buffer-soluble (50 mM CDTA-50 mM Ammonium oxalate extract) or alkali-soluble (4 M KOH extract) polysaccharides fractions are denoted by the suffix “-CDTA” and “-KOH”, respectively.

**Figure 7:**
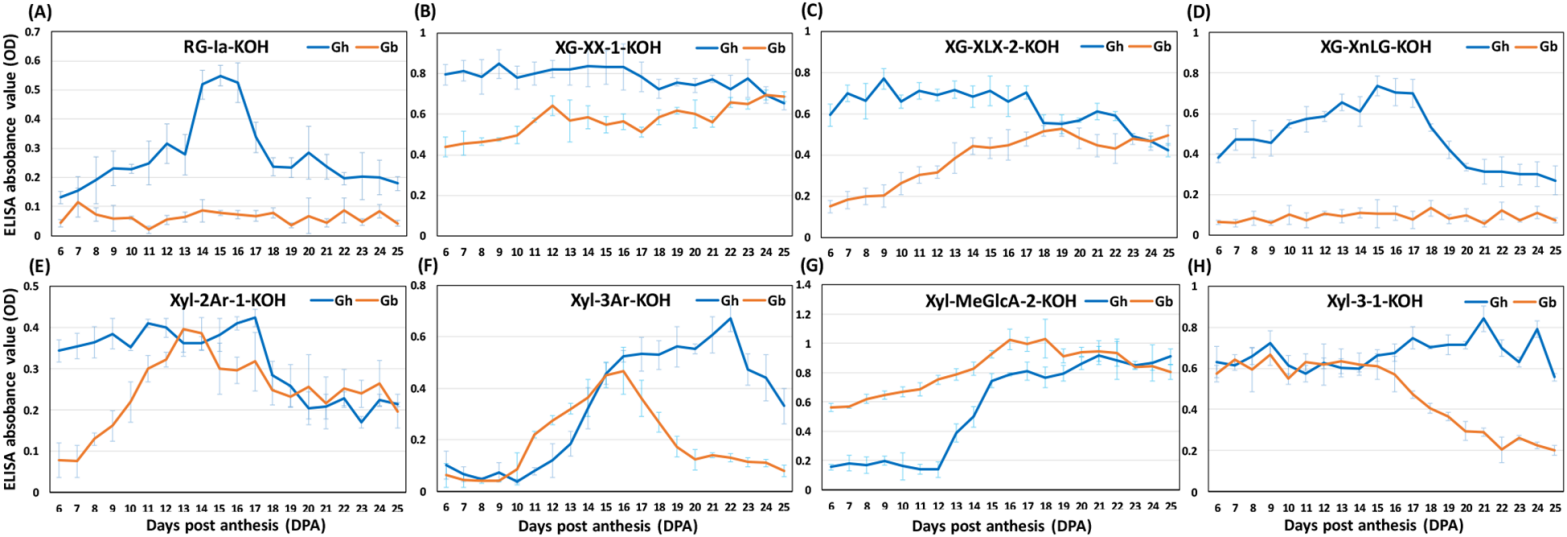
Glycome profiles of polysaccharide epitopes with different pattern between *Gh*and *Gb* fiber across development (6–25 DPA). **(A–H)**Representative profiles of polysaccharide epitopes listed in **Supplementary Data Sheet 8C** are shown here. The profiles of *Gh* (blue) and *Gb* (orange) show averaged optical density (OD) values of three biological replicates with standard error bars. Epitopes from alkali-soluble (4 M KOH extract) fractions are denoted by the suffix “-KOH”.

Both the SOM and heat maps provided complementary details of the epitope patterns and their differential distribution between the fibers of the two species during development (6–25 DPA). These data allowed us to group epitopes into three major “categories” (**Figures 5**–**7**; **Supplementary Data Sheet 8**), namely 1) polysaccharide epitopes that were similar in content and have no temporal variability between the two species, 2) polysaccharide epitopes that had similar profiles through all 20 DPAs, but varied in their content between the two species, and 3) polysaccharide epitopes that had more noticeable differences in their content and profiles between two species.

The first category included 44 polysaccharide epitopes with comparable content and similar profiles in the two species, indicating no significant difference in some of these epitopes between the fibers (**Figure 5**; **Supplementary Data Sheet 8A**). This group contained epitopes present in HGs (both methyl esterified and de-esterified), RG-I-BB (RG-I backbone epitopes), Gal4-BB (secondary galactan backbone of RG-I), highly branched RG-I (RG-I-Gal2, RG-I-Gal3s), XG (XG-(XXXG)2, XG-(XXXG)3, XG-L, XG-FF), and Xyl (Xyl-BBs, Xyl-GlcA, Xyl-MeGlcA-1). Most of the epitopes in this category, from both *Gh* and *Gb*, were found in the same SOM groups. For instance, buffer-soluble HG epitopes (HG-Bbde-1-C/-2-C, HG-BBMe-C) and RG-I epitopes (RG-I-BB-1-C/-2-C/-3-C, RG-I-Gal3-3-C) of both *Gh* and *Gb* were present in the same SOM group G9-C (**Figure 5**; **Supplementary Data Sheet 8A**). Similarly, some alkali-soluble HG epitopes (HG-Bbde-1-K, HG-Bbde-2-K), RG-I epitopes (RG-I-BB-1-K, RG-I-Gal2-1-K, RG-Ic-2-K), XG epitopes (XG-(XXXG)2-2-K, XG-FF-2-K, XG-Fuc-K, XG-L-1-K, XG-L-2-K), and Xyl epitopes (Xyl-BB3-K, Xyl-GlcA-K) from both species were assigned in the same G9-K SOM cluster. Many other polysaccharide epitopes from both species were also grouped in the same SOM groups, e.g., RG-I-BB-3-K, Gal4-BB3-C, Gal4-BB3-K, Gal4-BB6-K, RG-Ic-1-C, RG-I-Gal2-1-K, RG-I-Gal3-5-C, RG-I-Gal3-7-K, Xyl-MeGlcA-1-K, and Cal-K. SOM analysis revealed subtle differences in overall patterns and abundance of epitopes between the species. For example, many of the Xyl backbone epitopes (Xyl-BBs) from *Gh* were found in the G10-K SOM group, whereas the same epitopes from *Gb* were found in the neighboring G9-K SOM group, although profiles in these two SOM groups were not statistically different (**Figure 5**; **Supplementary Data Sheet 8A**).

The other 48 epitopes fell into the second category, which had the same profiles but showed significant differences in content between *Gh* and *Gb* (**Figure 6**; **Supplementary Data Sheet 8B**). Many of the highly branched RG-I, AGs, XG, and some Xyl epitopes were included in this group. Despite having similar profiles, these epitopes showed significant quantitative differences between the two species, and the SOM analysis effectively distinguished them based on their quantitative differences. For example, in *Gh*, six XG epitopes (XG-XX-1-C, XG-XX-2-C, XG-(XXXG)2-2-C, XG-(XXXG)3-2-C, XG-FF-2-C, XG-XLX-1-C) and three highly branched RG-I epitopes (RG-I-Gal3-1-C, RG-I-Gal3-2-C, RG-I-Gal3-7-C) were assigned to the G1-C group (**Figure 6**; **Supplementary Data Sheet 8B**). In contrast, the same above epitopes from *Gb* were distributed to the G2-Cand G4-C groups. Similarly, the G5-C group contained only polysaccharide epitopes from*Gh*, such as branched RG-I and XG epitopes, whereas the same epitopes from *Gb* were predominantly distributed to the G2-C and G10-C groups. Interestingly, only four epitopes out of 48 were present in higher amounts in *Gb* in comparison with *Gh*, whereas the remaining 44 epitopes showed higher presence in *Gh* relative to *Gb* (**Supplementary Data Sheet 8B**). Overall, glycome profiling showed that *Gb* had significantly lower levels of highly branched RG-I, AG, and Xyl epitopes compared with *Gh* (**Figure 5**; **Supplementary Data Sheet 8B**).

Comparative heat maps and SOM analysis clearly differentiated 14 polysaccharide epitopes with distinct profile patterns between the two species, forming a third category (**Figure 7**; **Supplementary Data Sheet 8C**). Interestingly, three epitopes, RG-Ia-K (present in the group G4-K for *Gh*, G12-K for *Gb*), XG-XnLG-K (present in G3-K for Gh, G12-K for Gb), and RG-I-Gal1-4-K (present in G4-K for *Gh*, G11-K for *Gb*), were not abundant and their profiles were flat in *Gb* through 6 to 25 DPA. On the contrary, in *Gh*, the profiles of these polysaccharide epitopes peaked in the middle of the fiber development. The content of five XG epitopes, XG-XX-1 (present in G6-K for *Gh*, G2-K for *Gb*), XG-XX-2 (present in G5-K for *Gh*, G2-K for *Gb*), XG-XLX-1 (present in G6-K for *Gh*, G2-K for *Gb*), XG-XLX-2 (in G2-K for *Gh*, G11-K for *Gb*), and XG-XLX-3 (in G1-K for *Gh*, G6-K were *Gb*), and two Xyl epitopes, Xyl-2Ar-1 (in G4-K for *Gh*, G8-K for *Gb*), and Xyl-2Ar-2 (in G3-K for *Gh*, G8-K for *Gb*) were lower in content in *Gb* at the earlier DPAs but increased gradually to match the amount of *Gh* at later stages (**Figure 7**; **Supplementary Data Sheet 8C**). A reverse profile was observed for the Xyl epitope Xyl-MeGlcA-2 (present in G2-K for *Gh* and G10-K for *Gb*), with lower content in *Gh* that gradually increased to equal the amount present in *Gb* at later DPAs. In *Gb*, the amount of two of the Xyl epitopes, Xyl-3Ar (present in G11-K for *Gh*, G8-K for *Gb*) and Xyl-3-1 (in G2-K for *Gh*, G3-K for *Gb*), was comparable to *Gh* at earlier stages but drastically reduced after 15 DPA to reach a significantly lower levels in *Gb* by 25 DPA.

About 34 epitopes were detected at very low levels and showed noisy profiles and therefore not analyzed further; these epitopes were mainly present in the SOM groups, G12-C and G12-K (**Figure 4**; **Supplementary Data Sheet 8D**).

### 3.4 Differentially accumulated transcript levels of glycosyltransferases between *Gh* and *Gb* fibers

#### 3.4.1 Differentially accumulated transcript levels of cellulose synthases

**Figure 8:**
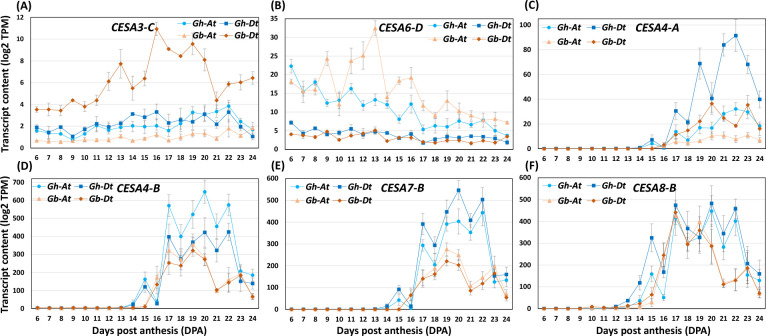
The profiles of cellulose synthase (*CESA*s) transcripts differentially accumulated in *Gh* and *Gb* fiber during development (6–24 DPA). **(A, B)** Transcript abundance profiles of PCW synthesizing *CESA*s. **(C–F)** Transcript abundance profiles of SCW synthesizing *CESA*s. Log2-transformed TPM values of A sub-genome (At) and D sub-genome (Dt) of both the species are shown in the plot.

Two types of CESAs, the PCW CESAs (CESAs 1/3/6) and SCW CESAs (CESAs 4/7/8), are known to beinvolved in the synthesis of cellulose microfibrils in PCW and SCW, respectively (Kim et al., 2019). Using *Arabidopsis* gene homology analysis, four *CESA1*, six *CESA3*, and 14 *CESA6*, and two each for *CESA4*, *CESA7* and *CESA8*, including both the A sub-genome (At) and D sub-genome (Dt), were identified in our cotton transcripts (**Supplementary Data Sheet 4**). Further, we compared the transcripts of these CESAs on a one-to-one basis. We found that the transcript levels of two of the PCW *CESA*s (*CESA3-C-Dt* and *CESA6-D-At*) and ten of the SCW *CESA*s (*CESA4-A-At* & *-Dt*, *CESA4-B-At* & *-Dt*, *CESA7-A-At* & *-Dt*, *CESA7-B-At* & *-Dt*, and *CESA8-B-At* & *-Dt*) (including both A and D genomes) were different between *Gh* and *Gb* (**Figure 8**; **Supplementary Data Sheet 4**). The most striking difference is that the transcript levels of PCW *CESAs* from *Gb* (*CESA3-C-Dt* and *CESA6-D-At*) was higher for a more extended period compared to *Gh*, whereas levels of all other PCW *CESAs* transcripts were similar in both species. In contrast, the ten differentially accumulated SCW *CESAs* showed higher transcript levels in *Gh* than in *Gb*. Most interestingly, there was a two to three-day delay in the expression of transcripts of all ten SCW *CESA*s in *Gb* fiber compared to *Gh*. In *Gh*, the transcript levels of SCW *CESAs* began to rapidly increase around 13 DPA, whereas in *Gb* this increase began around 15 DPA.

#### 3.4.2 Differentially accumulated transcript levels of xyloglucan synthesizing glycosyltransferases

**Figure 9:**
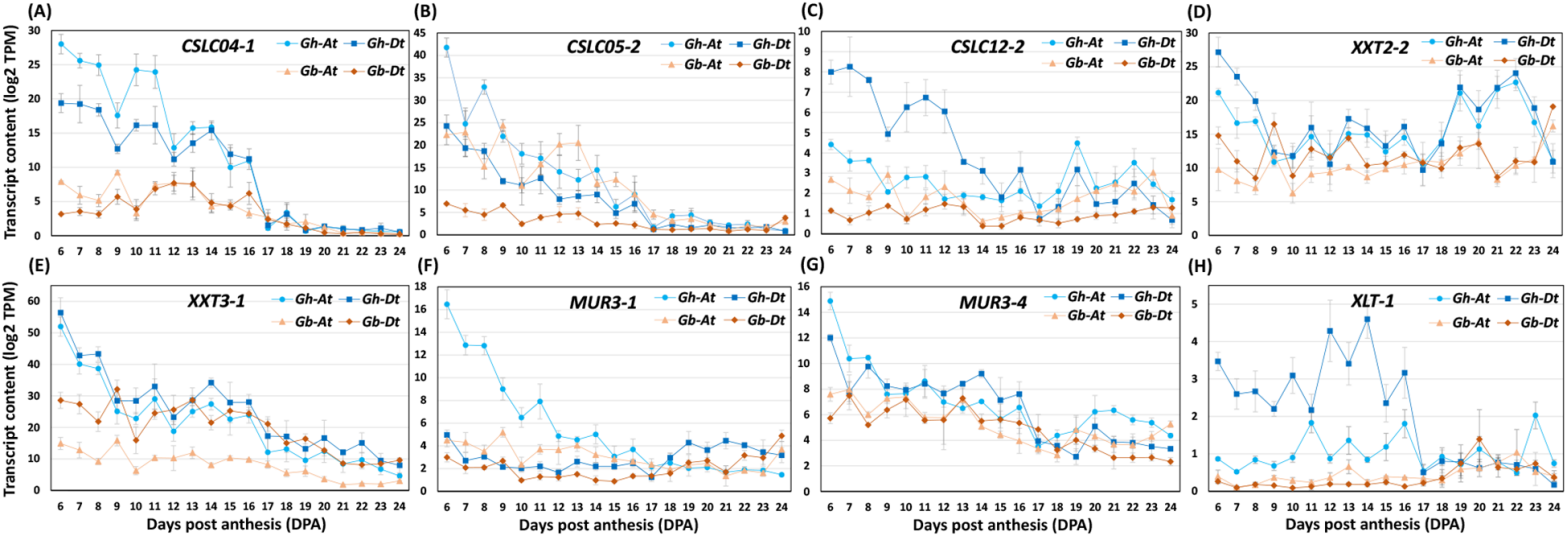
The profiles of XG-synthesizing glycosyltransferase transcripts differentially accumulated in *Gh* and *Gb* fiber during development (6–24 DPA). **(A–H)** Transcript abundance profiles of representative cellulose synthase-like-C enzymes (*CSLC*s); UDP-Xyl: XG α-1,6-xylosyltransferases (*XXT*s), and XG β-1,2-galactosyltransferases (*MUR3* and *XLT2*). Log2-transformed transcript per million (TPM) values of A sub-genome (At) and D sub-genome (Dt) of both the species are shown in the plot.

Next, we analyzed glycosyltransferases known to be involved in XG biosynthesis. Well-characterized XG-synthesizing enzymes from *Arabidopsis* are XG β-1,4-glucan synthases (cellulose synthase-like-C enzymes, CSLCs; CSLC4/5/6/8/12), XG α-1,6-xylosyltransferases (XXT1/2/3/4/5), XG β-1,2-galactosyltransferases (MUR3 and XLT2), and an α-1,2-L-fucosyltransferase (FUT1) (**Supplementary Figure 1**) (Julian and Zabotina, 2022). The CSCLs involved inthe synthesis of the glucan backbone: XXTs add xylose residues to the specific glucoses, MUR3/XLT2galactosylates specific xyloses, and FUT1 fucosylates the galactose residues. By homology search, we identified 16 *CSLC*s, 10 *XXT*s, eight *MUR3*s, four *XLT2*s, and four *FUT1*s in both the A and D sub-genomes of cotton (**Supplementary Data Sheet 4**; Swaminathan et al., 2024). Most importantly, the comparative analysis of *Gh* and *Gb* across both the A and D genomes showed that all the differentially accumulated XG-synthesizing enzymes had higher transcript levels in *Gh* than in *Gb* (**Figure 9**; **Supplementary Data Sheet 4**). The differentially and highly expressed glycosyltransferases in *Gh* were five *CSLC*s (*CSLC04-1At* & *-1Dt, CSLC05-2At & -2Dt, CSLC12-2Dt*), four *XXT*s (*XXT2-2At* & *-2Dt, XXT3-1At* & *-1Dt*), three *MUR3*s (*MUR3-1At, MUR3-4At* & *-4Dt*), and two *XLT*s (*XLT-1At* & *-1Dt*).

#### 3.4.3 Differentially accumulated transcript levels of xylan synthesizing glycosyltransferases

Xyl-synthesizing glycosyltransferases identified so far are Xyl β-1,4-xylosyltransferases (IRXs; irregular xylem), Xyl α-glucuronyltransferases (GUXs), glucuronoxylan methyltransferase-like proteins (GXMT), and Xyl arabinosyltransferases (XATs) (**Supplementary Figure 1**) (Smith et al., 2017; Chen et al., 2020). IRXs are responsible for Xyl backbone synthesis. GUXs areinvolved in adding α-1,2-D-glucuronic acid (GlcA) to the Xyl backbone, and GXMTs methylate glucuronic acids in the side chains of Xyl. XATs are involved in adding arabinose residues to Xyl backbones, while O-acetyltransferases (ESK/RWA/TBL) acetylate the backbone. In addition, there are other enzymes proposed to synthesize the specific reducing end oligosaccharide of the Xyls (FRA8-1/PARVUS/IRX8), and the formation of which leads to the termination of Xyl backbone elongation (Chen et al., 2020). Homology analysis revealed 30 *IRXs*, 16 *GUXs*, 22 *GXMTs*, 40 O-acetyltransferases (*ESK/RWA/TBL*) and 14 glycosyltransferases predicted to produce enzymes that synthesize the reducing end of Xyl (*FRA8-1/PARVUS/IRX8*), from both A and D cotton genomes (**Supplementary Data Sheet 4**).

**Figure 10:**
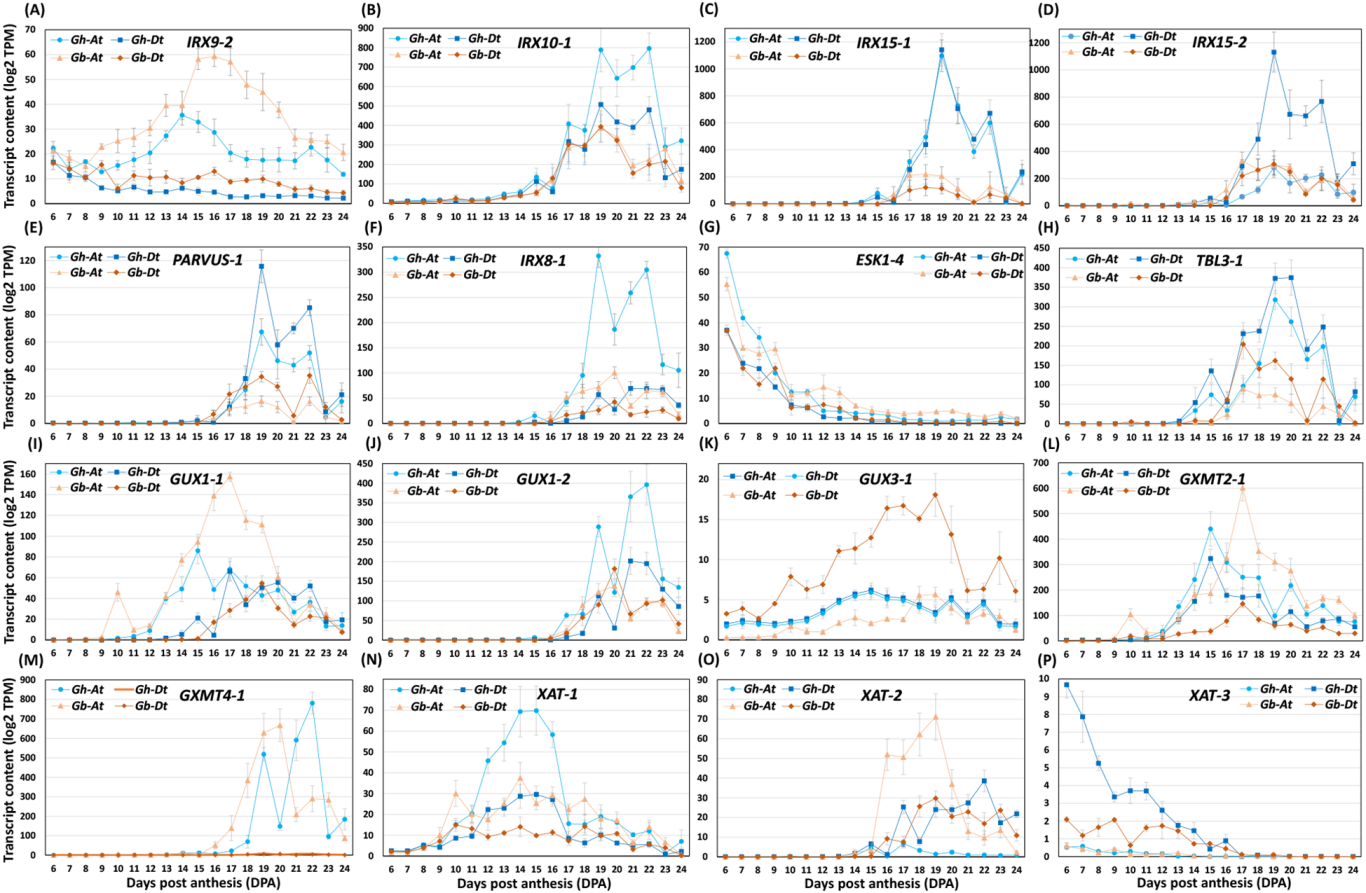
The profiles of Xyl-synthesizing glycosyltransferase transcripts differentially accumulated in *Gh* and *Gb* fiber during development (6–24 DPA). **(A–P)** Transcript abundance profiles of representative Xyl β-1,4-xylosyltransferases (*IRX*s; irregular xylem), O-acetyltransferases (*ESK*/*TBL*), reducing end synthesizing glycosyltransferase (*PARVUS*), UDP-GlcA: xylan α-glucuronyltransferases (*GUX*s), glucuronoxylan methyltransferase-like proteins (*GXMT*), and xylan arabinosyltransferases (*XAT*s). Log2-transformed TPM values of A sub-genome (At) and D sub-genome (Dt) of both the species are shown in the plot.

Comparative transcript analysis of Xyl backbone-synthesizing glycosyltransferases demonstrated that 17 out of 30 *IRX*s (*IRX9-3Dt, IRX10-1At* & *-1Dt, IRX10-2At* & *-2Dt, IRX10-3At* & *-3Dt, IRX14-1At* & *-1Dt, IRX14-2At* & *-2Dt, IRX15-1At* & *-1Dt, IRX15-2Dt, IRX15-3At* & *-3Dt, IRX15-4Dt*) were present in higher amount in *Gh* in comparison with *Gb* (**Figure 10**; **Supplementary Data Sheet 4**). However, only two *IRX*s (*IRX9-2At* & *-2Dt*) were found to have higher transcript levels in *Gb* than in *Gh*. Ten O-acetyltransferases (*ESK1-1At* & *-1Dt, RWA1-1Dt, RWA1-3At, RWA1-4At* & *-4Dt, TBL3-1At* & *-1Dt, TBL3-2At* & *-2Dt*) were more highly expressed in *Gh* than in *Gb*, while only one was highly expressed in *Gb* (*TBL30-3Dt*) than in *Gh*. Among the enzymes proposed to synthesize the reducing end oligosaccharide in Xyl, ten glycosyltransferases (*FRA8-1Dt, PARVUS-1At* & *-1Dt, PARVUS-2At* & *-2Dt, PARVUS-3At, PARVUS-4Dt, IRX8-1At, IRX8-2At* & *-2Dt*) showed higher transcript levels in *Gh* relative to *Gb*. Among the enzymes involved in branching of the Xyl backbone, the transcript levels of two *GUX*s (*GUX1-2At, GUX2-1At*) were higher in *Gh*, whereas four were higher in *Gb* (*GUX1-1At, GUX2-2At* & *-2Dt, GUX3-1Dt*). Among *GXMT*s, the levels of four transcripts were higher in *Gh* (*GXMT4-3At* & *-3Dt, GXMT4-4At* & *-4Dt*), whereas three were higher in *Gb* (*GXMT2-1At, GXMT5-2At* & *-2Dt*). Among *XAT*, the levels of two transcripts were higher in *Gh* (*XAT-1At, XAT-3Dt*), and only one was higher in *Gb* than in *Gh* (*XAT-2At*). Overall, the results indicate that Xyl backbone-synthesizing glycosyltransferases with higher transcript levels were more numerous in the *Gh* genome than in the *Gb* genome. However, the number of glycosyltransferases with higher transcript levels that contribute to Xyl side chain residues was equal in the genome of both cotton species.

#### 3.4.4 Differentially accumulated transcript levels of homogalacturonan synthesizing glycosyltransferases

Synthesis of HG is carried out by galacturonosyltransferase (GAUTs) and galacturonosyltransferase-like (GATLs) enzymes, which add galacturonic acid (GalA) residues to the growing chain of HG backbones. The methyl and acetyl groups are added to the HG backbone by methyl transferases (CGRs/QUAs) and O-acetyltransferases (TBLs/TBRs), respectively (Atmodjo et al., 2013; Engle et al., 2022) (**Supplementary Figure 1**).

**Figure 11:**
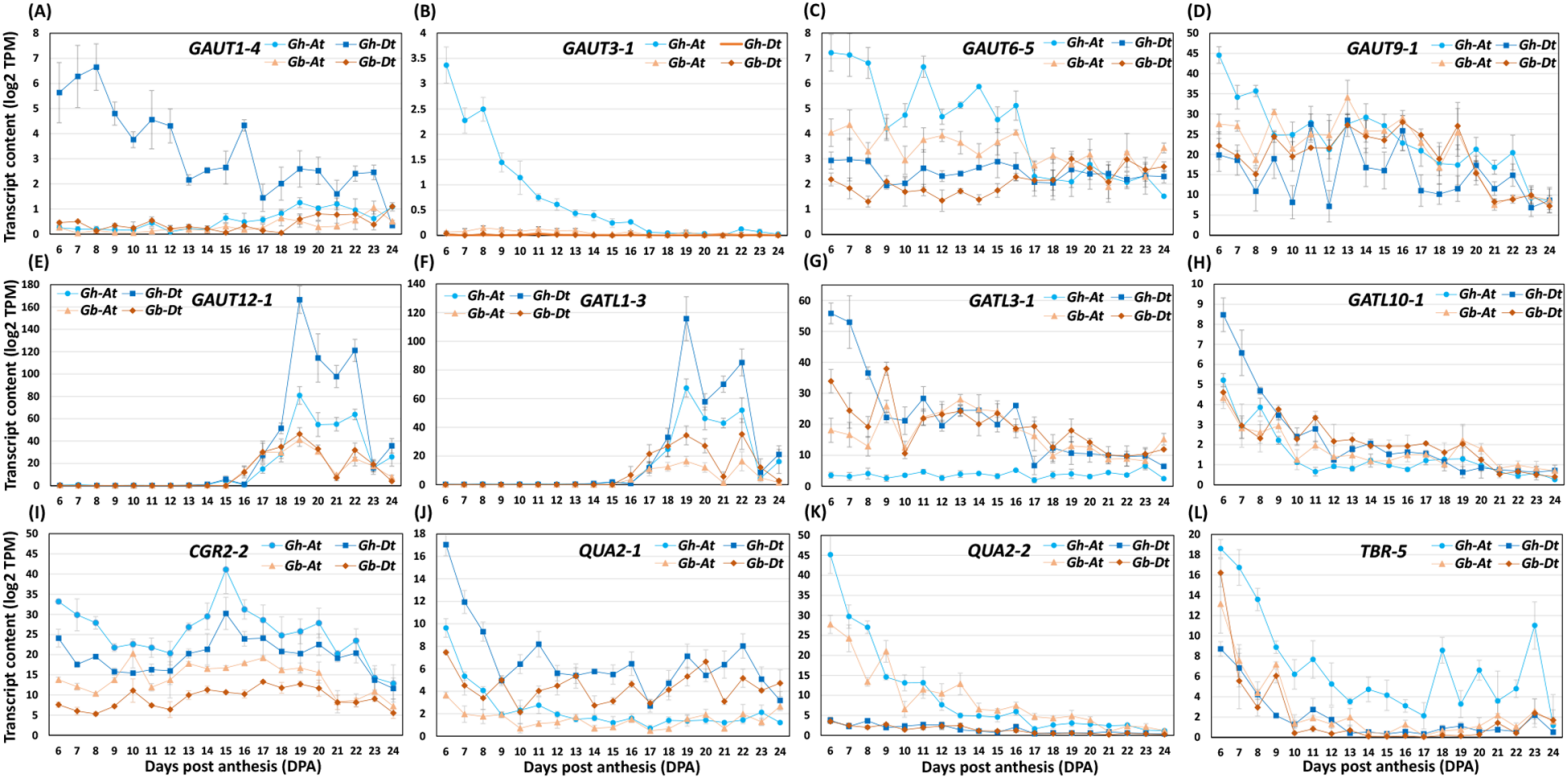
The profiles of HG-synthesizing glycosyltransferase transcripts differentially accumulated in *Gh* and *Gb* fiber during development (6–24 DPA). **(A–L)** Transcript profiles of representative galacturonosyltransferases (*GAUT*s), galacturonosyl transferase-like enzymes (*GATL*s), methyltransferases (*CGR*s/*QUA*s), and acetyltransferases (*TBR*s). Log2-transformed TPM values of A sub-genome (At) and D sub-genome (Dt) of both the species are shown in the plot.

Homology search of HG-related glycosyltransferases revealed that there are 48*GAUTs*, 24 *GALTs*, 24 methyltransferases(*CGRs/QUAs*), and 14 acetyltransferases (*TBLs/TBRs*) genes found in cotton fibers (**Supplementary Data Sheet 4**; Swaminathan et al., 2024). Comparative transcript analysis showed that the transcript levels of ten *GAUT*s were higher in *Gh* than in *Gb* (*GAUT1-4Dt, GAUT3-1At, GAUT4-1Dt, GAUT6-5At, GAUT8-2Dt, GAUT9-1At, GAUT12-1At* & *-1Dt, GAUT12-2At* & *-2Dt*), whereas the levels of four were higher in *Gb* (*GAUT7-1Dt, GAUT8-1Dt, GAUT15-1At* & *-1Dt*) (**Figure 11**; **Supplementary Data Sheet 4**). Among *GATL*s, the transcript levels of ten were higher in *Gh* (*GATL1-3At* & *-3Dt, GATL1-4At* & *-4Dt, GATL1-5At* & *-5Dt, GATL2-2Dt, GATL3-1Dt, GATL7-2Dt, GATL10-1Dt*), whereas only one was higher in *Gb* (*GATL2-3Dt*). Among methyl transferases, the transcript levels of six were higher in *Gh* (*CGR2-2At* & *-2Dt, CGR2-4At, QUA2-1Dt, QUA2-2At, QUA3-1At*) and no methyl transferase transcripts were higher in *Gb*. Among acetyltransferases, the transcript levels of five were higher in *Gh* (*TBL3-1At* & *-1Dt, TBL3-2At* & *-2Dt, TBR-5At*) and only one was higher in *Gb* (*TBR-1Dt*) (**Figure 11**; **Supplementary Data Sheet 4**).

#### 3.4.5 Differentially accumulated transcript levels of rhamnogalacturonan-I and arabinogalactan synthesizing glycosyltransferases

RG-I is a highly branched pectin that has a complex structure. RG-I primary backbone is branched with the β-1,3-galactans (rarely found), β-1,4-galactans, and α-1,5-arabinans side chains. Further, the β-1,4-galactan side chain is decorated with β-1,6-linked galactans and arabinan side chains (**Supplementary Figure 1**) (Atmodjo et al., 2013; Amos et al., 2022). RG-I:Galacturonosyltransferases (RG-I:GalATs) andRG-I:rhamnosyltransferases (RRTs) are known to synthesize the primary backbone of RG-I in *Arabidopsis*. The AG-GALTs and β-1,4-galactan synthases (GALSs) synthesize β-1,3-galactan and β-1,4-galactan side chains of RG-I, respectively. The arabinan α-1,5-L-arabinosyltransferases (ARAD) add the arabinan side chain to the RG-I backbone. Further, the β-1,6-galactosyltransferases (GALT29A/GALT31A) and β-1,3-glucuronosyl transferases (GlcAT14) are involved in decorating side chains of RG-I with β-1,6-linked galactans, and β-1,3-linked glucuronic acid (could be part of AG proteins of CW), respectively (Showalter and Basu, 2016). Homology searching of genes involved in RG-I biosynthesis showed that there were eight *RG-I:GalATs*, 34 *RRTs*, six *GALSs*, 34 *AG: GALTs*, 10 *GALT29A/GALT31As*, and four *ARAD*s expressed in cotton fibers, from both A and D homeologs (**Supplementary Data Sheet 4**).

**Figure 12:**
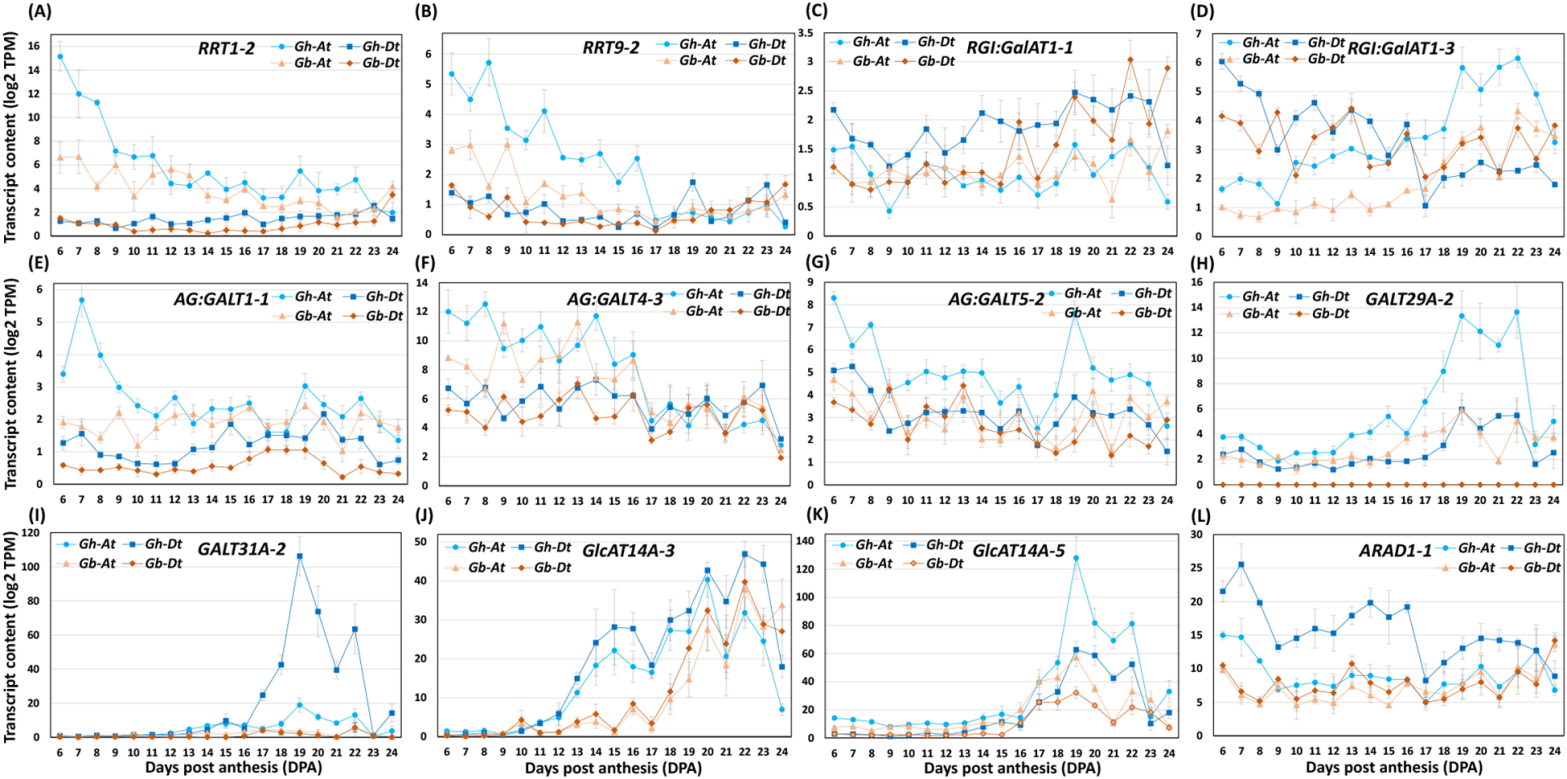
The profiles of RG-I-synthesizing glycosyltransferase transcripts differentially accumulated in *Gh* and *Gb* fiber during development (6–24 DPA). **(A-L)** Transcript profiles of representative RG-I:rhamnosyltransferases (*RRT*s), galacturonosyl transferases (*RG-I:GalAT*s), β-1,6-galactosyltransferases (*GALT29A*/*GALT31A*/*AG: GALT*s), β-1,6-glucuronosyl transferases (*GlcAT*), and arabinan α-1,5-L-arabinosyltransferase (*ARAD*). Log2-transformed TPM values of A sub-genome (At) and D sub-genome (Dt) of both the species are shown in the plot.

Comparative transcriptome analysis of RG-I synthesizing glycosyltransferases revealed that the transcript levels of three *RG-I:GalAT*s were higher in *Gh* (*RGI: GalAT1-1Dt, RGI: GalAT1-3At* & *-3Dt*) than in *Gb* and none were higher in *Gb* in comparison with *Gh* (**Figure 12**; **Supplementary Data Sheet 4**). Among *RRT*s, the transcript levels of three were higher in *Gh* (*RRT1-2At, RRT4-1Dt, RRT9-2At*) and two were higher in *Gb* (*RRT1-4Dt, RRT9-1At*). Out of all *GALS*, the transcript levels of one *GALS2-1Dt* was higher in *Gh*, while the transcript levels of another *GALS1-2Dt* was higher in *Gb*. Among *AG: GALT*s, the transcript levels of five were higher in *Gh* (*AG: GALT1-1At, AG: GALT4-3At, AG: GALT5-2At, AG: GALT5-3Dt, AG: GALT8-2At*), and only *AG: GALT1-2Dt* had a higher transcript level in *Gb*. Among *GALT29As/GALT31As*, the transcript levels of five genes, *GALT29A-1At, GALT29A-2At, GALT31A-1At* & *-1Dt, GALT31A-2Dt* were higher in *Gh* in comparison with *Gb* and none in *Gb*. Among all *GlcAT14*s, the transcript levels of eight genes were higher in *Gh* (*GlcAT14A-1At* & *-1Dt, GlcAT14A-3At* & *-3Dt, GlcAT14A-4At, GlcAT14A-5At* & *-5Dt, GlcAT14C-1Dt*) and none were higher in *Gb*. Among *ARAD*s, the transcript levels of two were higher in *Gh* (*ARAD1-1At* & *-1Dt*), and only *ARAD1-2Dt* was higher in *Gb* than in *Gh* (**Figure 12**; **Supplementary Data Sheet 4**).

#### 3.4.6 Differentially accumulated transcript levels of expansins

**Figure 13:**
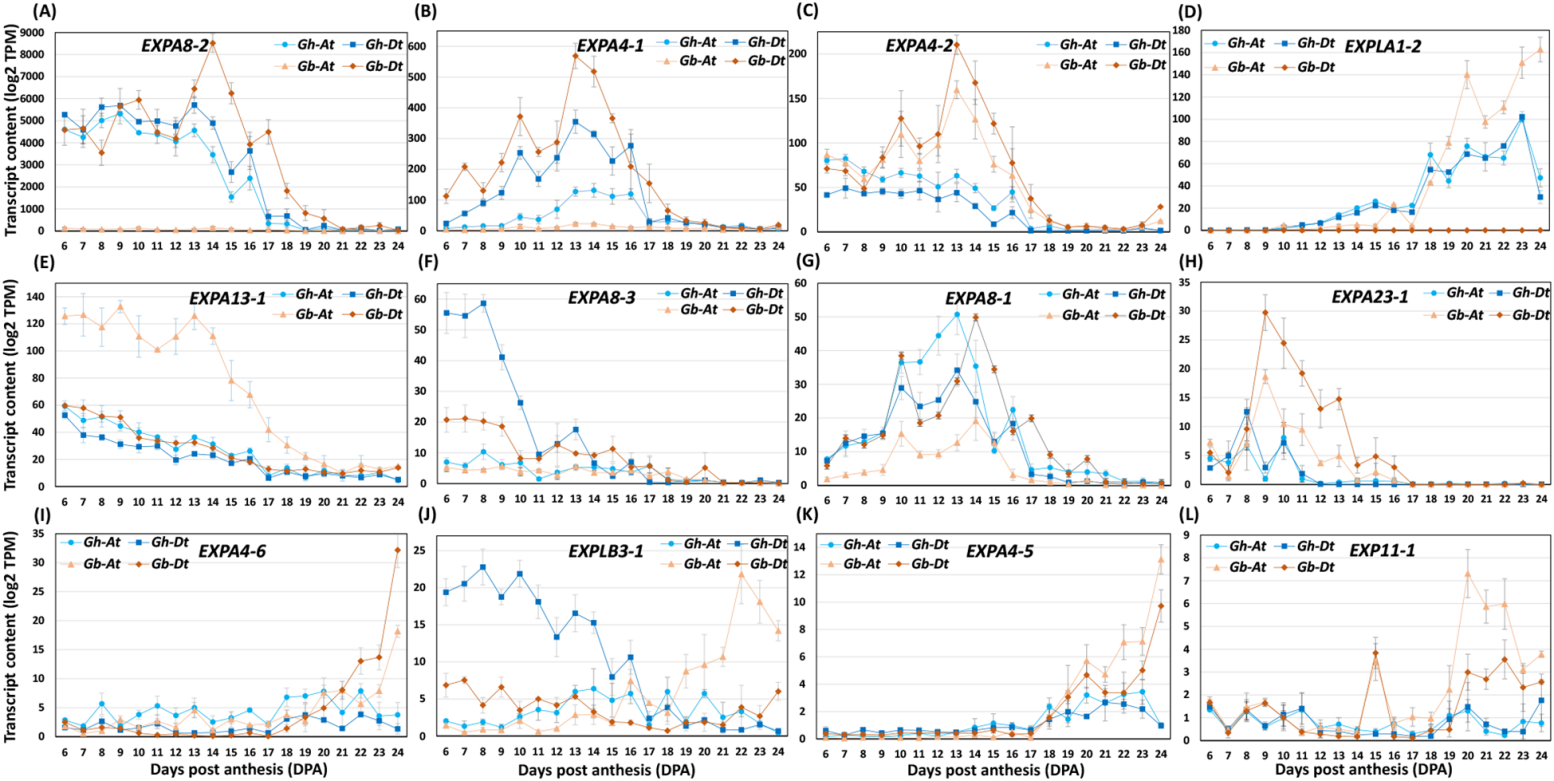
The profiles of expansins transcripts differentially accumulated in Gh and Gb fiber duringdevelopment (6 - 24 DPA). **(A–L)** Profiles of differentially accumulated transcript levels of expansins. Log2-transformed TPM values of A sub-genome (At) and D sub-genome (Dt) of both the species are shown in the plot.

Recent studies highlighted the importance of CW-loosening protein expansin as one of the major players in fiber elongation (Sampedro and Cosgrove, 2005; Xu et al., 2013; Li et al., 2016; Lv et al., 2020). We compared expansin transcript levels in both *Gh* and *Gb* fibers. Levels of many of the expansion transcripts were higher in *Gb* than in *Gh* (**Figure 13**; **Supplementary Data Sheet 9**). Thus, the transcripts level of 14 expansins (*EXPA4-1Dt, EXPA4-2At* & *-2Dt, EXPA4-3At, EXPA4-5At* & *-5Dt, EXPA4-6At* & *-6Dt, EXPA8-2Dt, EXPA13-1At, EXPLB3-1At, EXPLA1-2At, EXP11-1At* & *-1Dt*) were higher in *Gb*, whereas eight (*EXPA4-4At* & *-4Dt, EXPA8-2At, EXPA8-3Dt, EXPA15-1At, EXPA15-2Dt, EXPLB3-1Dt, EXPLA1-2Dt*) were higher in *Gh* relative to *Gb*. In *Gb*, the transcript levels of eight of the expansins (*EXPA4-5At* & *-5Dt, EXPA4-6At* & *-6Dt, EXPLB3-1At, EXPLA1-2At, EXP11-1At* & *-1Dt*) rapidly increased after 12 DPA and were present at higher levels at later DPAs. In the case of *Gh*, only two transcripts (*EXPLA1-2At* & *-2Dt*) showed similar patterns, but their content levels were significantly lower than in *Gb* (**Figure 13**; **Supplementary Data Sheet 9**).

## 4 Discussion

The two most cultivated cotton species, *G. hirsutum* (*Gh*) and *G. barbadense* (*Gb*), exhibit distinct fiber properties and well-defined, overlapping developmental stages. These species offer an excellent opportunity to study the dynamic temporal remodeling and development of a fiber cell that defines fiber characteristics, and comparative analysis between the species represents a natural form of experimental perturbation that can inform our understanding of the molecular underpinnings of the mature phenotypes. Earlier studies pointed out that cotton fiber development includes tightly controlled complex gene expression networks, biosynthetic pathways, physiology, and development that eventually result in dynamic sequential changes in fiber CW polysaccharide epitopes, which determine the fiber quality in different species of cotton (Haigler et al., 2012; Jan et al., 2022; Jareczek et al., 2023). The primary focus of our study was to analyze the fiber from both *Gh* and *Gb*, identify critical polysaccharides that drive fiber quality traits, and identify potential glycosyltransferases that may be responsible for the synthesis of these polysaccharides.

The uniqueness and power of this large-scale comparative study lie in the simultaneous profiling of the glycome, and transcriptome, on fibers from two different species, collected daily for 20 successive days (6 to 25 DPA), and grown under controlled conditions. Our previous large-scale glycome and transcriptome study on *Gh* fiber identified critical polysaccharides and several key putative glycosyltransferases that contribute to important fiber traits (Swaminathan et al., 2024). Although in this present study proteomic analysis was conducted only on *Gb* fiber, the integration of multi-omics data demonstrated correlation between the transcript levels and the corresponding glycosyltransferases and other polysaccharide-synthesizing enzymes detected in the microsome-enriched proteome fraction (**Figure 2**). However, the correlation between the transcript levels and the protein contents was limited (only about 38%), because the accumulation, turnover and activity of glycosyltransferases may dynamically vary based on their synthesis and degradation. Overall, some of the significantly correlated glycosyltransferases may be involved in the formation of specific polysaccharide epitopes during fiber development.

Another important aspect of our study was that these 20 days (6–25 DPA) were selected to cover the most critical stages of fiber development, including fiber elongation, transition, and initial SCW thickening stages (Haigler et al., 2012). These stages of development were also characterized by distinct gene expression pattern between *Gh* and *Gb*, which coincided with CW remodeling that potentially led to differential fiber development between *Gh* and *Gb*, resulting in different fiber quality (Al-Ghazi et al., 2009; Liu et al., 2023). This comparative analysis, using coordinated analyses of the transcriptome, proteome, and glycome, represents a promising approach to understanding how the subtle details of CW polysaccharide alterations during key stages of CW synthesis might predict the underlying molecular machinery.

### 4.1 Differences in cellulose accumulation between *Gh* and *Gb* fibers

Here, we observed a three-day delay in the rapid accumulation of cellulose content in *Gb* fibers compared to *Gh* during the transition period prior to SCW thickening (**Figure 1**). This observation agrees with earlier studies reporting an extended fiber elongation phase at *Gb* compared to *Gh* (Tuttle et al., 2015; Hu et al., 2019; Pettolino et al., 2022). Such a delay in cellulose accumulation has been reported previously, even across different *Gb* accessions and growing conditions (Lee et al., 2015; Pettolino et al., 2022). Coincidentally, a similar delay in transcript accumulation was observed for ten SCW *CESA*s in *Gb* compared to *Gh* (**Figure 8**). In addition, transcript levels of two PCW *CESA*s in *Gb* were higher and persisted for a longer period than in *Gh*. Previous transcriptomic studies utilizing only three time points (10, 15, 20 DPA) revealed that only one of the PCW *CESA* (*CESA6*) had a higher transcript level in *Gb* relative to *Gh* (Hernandez-Gomez et al., 2015). Several other studies that examined fewer time points of fiber development also reported lower transcript levels and a few days’ delay in the accumulation of transcripts in some SCW *CESA*s in *Gb* relative to *Gh* (Lacape et al., 2012; Li et al., 2013; Hernandez-Gomez et al., 2015).

It was proposed that the delayed timing of cellulose accumulation might be one of the possible reasons for the better fiber quality of *Gb* relative to *Gh* (Avci et al., 2013; Lacape et al., 2012; Li et al., 2013; Rajasundaram et al., 2014; Pettolino et al., 2022). Additionally, it was well established that higher SCW *CESA* transcript accumulation leads to increased cellulose accumulation at the SCW developmental stage, resulting in the thickening/rigidification of the CW, which in turn halts CW expansion (Haigler et al., 2012). Our findings, combined with those of earlier reports, suggest that slower accumulation of cellulose (due to extended/higher PCW *CESA*s transcript accumulation combined with delayed lower levels of transcript accumulation of SCW *CESA*s for a few days) might have resulted in delayed rigidification of CW, which potentially resulted in longer period of elongation, and longer and thinner fibers of *Gb*. Even though cellulose accumulation was delayed by about three days and had a lower content in *Gb* at around 19 DPA compared to *Gh* (**Figure 1**), cellulose accumulation in *Gb* increased rapidly later. By the end of 25 DPA, both species had reached the same level of cellulose. Earlier, Li et al. (2013) reported that the *CESA8* was the major player for SCW cellulose accumulation in both *Gh* and *Gb* fiber and significantly contributed to rapid cellulose accumulation at later stages in *Gb* during the SCW thickening stage. Interestingly, our transcriptome data also showed that the transcript level of only *CESA8-B* (**Figure 8F**) was highest and equal in both species in comparison to all other SCW *CESA*s (**Figure 8**; **Supplementary Data Sheet 4**). However, the amount of cellulose accumulated per unit fiber length might be lower in *Gb* compared to *Gh*, as reported earlier (Avci et al., 2013), and it might have resulted in longer fiber elongation time and longer and thinner fiber in *Gb*. In our future study, we will investigate fiber length, growth rate, cellulose accumulation and microfibril orientation to relate them to fiber quality traits.

Some unanswered questions remain in order to understand the whole dynamics and contribution of CESAs to fiber quality traits, such as 1) Would the temporal patterns of transcripts of *CESA*s that we observed directly correlate with protein/enzyme level and dynamics? 2) How much do the differentially accumulated PCW and SCW *CESA*s contribute to cellulose crystallinity? 3) What is the role of PCW and SCW crystalline cellulose in temporal plasticity, rigidity, elongation, and strength of fiber in both species? 4) How does the cellulose in PCW and SCW interact with other CW components and thus contribute to fiber quality?

### 4.2 Differences in matrix polysaccharides and differentially accumulated transcript levels of glycosyltransferases that could contribute to differences in fiber quality between *Gh* and *Gb*

Comparative analysis of the polysaccharide composition and related glycosyltransferases between *Gh* and *Gb* fiber revealed many interesting details that might be the cause for the fiber quality differences between these cotton species. First, it is worth pointing out the differences in the total amount of buffer-soluble and alkali-soluble polysaccharide fractions. In *Gh*, the content of buffer-soluble polysaccharides was higher than that of alkali-soluble polysaccharides at 6–7 DPAs, whereas in *Gb* the content of these two fractions were more comparable (**Figure 1**). However, the level of buffer-soluble polysaccharides reduced more rapidly in *Gh* than in *Gb* during development, and at later stages however, the relative proportion of buffer-soluble polysaccharides remained at the same level in the two species. These differences reflected in the differences in polysaccharide epitope distribution, discussed below.

Quantitative heat maps and SOM analyses of polysaccharide epitope profiles revealed significant differences between the two species, which guided a comparative analysis of the glycome and glycosyltransferase transcriptome data. The most interesting aspect of our findings was that some of the same polysaccharide epitopes from *Gh* and *Gb* fell into two different SOM groups, which suggests that polysaccharide distribution differs in fiber CW between the two species. Although some of these particular differences appeared insubstantial and statistically insignificant, even subtle differences, taken together, could potentially impact fiber growth and development. In particular, the polysaccharide epitopes from the second and third categories (**Figures 6**, **7**) may be promising targets for further detailed investigations.

Our large-scale glycome profiling showed that most of the buffer and alkali-extracted fucosylated and non-fucosylated XG epitopes were very low in *Gb* in comparison with *Gh* (**Figures 6**, **7**), which agrees with similar earlier comparative studies (Avci et al., 2013; Hernandez-Gomez et al., 2017; Guo et al., 2019). These earlier studies reported that the presence of XG was lower in cotton fiber middle lamellae (CFML, the special adhesive outer layer of PCW) and PCW from *Gb* than in *Gh*. The authors proposed that the role of CFML is to adhere to the adjacent fibers, which facilitates the formation of organized tissue-like fiber bundles around each seed. This orderly fiber packing facilitates the elongation of fibers to a maximum within the confined space inside each locule of the cotton boll (Singh et al., 2009). However, Avci et al. (2013) reported that CFML was not required for fiber elongation in *Gb*, since it continued to elongate rapidly even after lysis of CFML. Additional studies will be required to confirm the correlation between the amount of XG and the dynamics of CFML/PCW development in promoting fiber length in different species of cotton.

Interestingly, in our transcriptome profiling study, we found that the transcript levels of all glycosyltransferases related to XG biosynthesis (*CSLCs/XXTs/MUR3s/XLT*) (**Figure 9**; **Supplementary Data Sheet 4**) were lower in *Gb* than in *Gh*, which might have resulted in lower XG enzymatic activity and consequently less XG epitope content in *Gb* than in *Gh*. A previous study by Guo et al. (2019) using fiber at 10, 15, and 20 DPA showed that the transcript levels of a *CSLC4* and a *MUR3* were lower in *Gb* than in *Gh*. In our transcriptome data, we found that the transcripts of a significantly larger set of various XG-synthesizing glycosyltransferases from both At and Dt genomes accumulated less in *Gb* compared to *Gh*. It will be interesting in future studies to examine the contribution of all these enzymes to the fiber quality in *Gb*.

It was presumed previously that XG interacts with cellulose microfibrils through non-covalent hydrogen bonds that could potentially contribute to rigidity to the CW and restrain cell expansion (Cosgrove, 2022; McCann et al., 1990; Somerville et al., 2004; Park and Cosgrove, 2015; Pękala et al., 2023). Interestingly, some of the earlier studies showed that the short fiber mutants (*Ligon lintless-1 & 2*; *Li_1_/Li_2_*) had a higher accumulation of XG content during elongation stages due to higher expression of XG-synthesizing glycosyltransferases, which presumably resulted in an extreme reduction in fiber length (Shao et al., 2011; Naoumkina et al., 2017). Overexpression of a MUR3 (GhMUR3-2Dt; Gohir.D10G136900) protein in *Gh* cotton resulted in shorter and thicker fibers compared to wild type (Wu et al., 2024). All data obtained so far suggests that a higher amount of XG negatively correlates with fiber length, most likely impacting the “xyloglucan-cellulose interaction”, which may lead to an increase in the rigidity of fiber CWs. Thus, the results suggest that a lower amount of XG seems to loosen this interaction, which might have helped extend the elongation time and promote longer fibers in *Gb* in comparison with *Gh*.

Another hemicellulosic polysaccharide, xylans (Xyl), is the major component in SCWs, and due to its interaction with cellulose and lignin, Xyl is essential for SCW architecture and strength (Gille and Pauly, 2012). In our previous study, we demonstrated that some of the Xyl epitopes (Xyl-3Ar, Xyl-MeGlcA) and transcripts of associated glycosyltransferases rapidly peak at the transition stage in *Gh* fiber before the rapid SCW cellulose accumulation/CW thickening and the changes in microfibril orientation (Swaminathan et al., 2024). Here, we found that the amount of loosely bound buffer-extractable Xyls, mostly represented by the Xyl backbone epitopes (Xyl-BB) and a glucuronoxylan epitope (Xyl-GlcA), was lower in *Gb* than in *Gh* (**Figure 6**) and might contain undetectable Xyls with a high degree of substitution. The alkali-extractable arabinoxylans (Xyl-2Ar, Xyl-3Ar) and a methylated glucuronoxylan (Xyl-MeGlcA-2) showed different profiles between *Gh* and *Gb* (**Figure 7**), and Xyl-MeGlcA-2 was present in higher amounts in *Gb* than in *Gh*. Somewhat similar differences in Xyl-related epitope profiles were reported earlier (Avci et al., 2013; Hernandez-Gomez et al., 2015).

We compared the transcript levels of Xyl-synthesizing glycosyltransferases between the two species. Transcript levels of most of the *IRXs/FRA8/PARVUSs* and *ESKs/TBLs/RWAs* genes, which produce enzymes that are involved in Xyl backbone synthesis and decorating Xyl backbone with acetyl/methyl groups, respectively, were higher in *Gh* than in *Gb*. This correlates well with the presence of higher amounts of Xyl backbone epitopes in *Gh* than in *Gb* (**Figure 10**). On the other hand, the transcript levels of some of the *GUXs* and *GXMTs*, which are responsible for the synthesis of methylated glucuronoxylans, were higher in *Gb* than in *Gh*, which correlates with the higher content of Xyl-MeGlcA-2 epitopes detected in *Gb* (**Figure 7**).

Recent reverse-genetic studies in cotton with some of the Xyl-synthesizing glycosyltransferases (*FRA8*/*IRX9*/*IRX10*/*IRX14*/*IRX15*) from *Gh* showed that Xyls play an important role in cellulose microfibril orientation, SCW cellulose deposition, fiber length, and thickness (Li et al., 2014; Chen et al., 2020; Guo et al., 2023; Li et al., 2024). In our previous large-scale profiling study (Swaminathan et al., 2024), we also observed that three heteroxylan epitopes were highly correlated with the cellulose content, the transcript levels of SCW *CESA*s, the cellulosic microfibril orientation and the CW thickness phenotype of *Gh* fiber. The function of heteroxylans (glucuronoxylans and arabinoxylans) is usually related to the strengthening of CW by acting as a guiding scaffold for cellulose microfibril orientation/arrangement (Grantham et al., 2017; Smith et al., 2017; Crowe et al., 2021; Pfaff et al., 2024). The strength of the cotton fiber CW is an important quality essential for the textile industry processing. The differential profiles and contents of methylated glucuronoxylan (Xyl-MeGlcA) and arabinoxylans (Xyl-2Ar and Xyl-3Ar) observed in *Gb* could potentially contribute to its stronger fiber. In our proteome data, we also noticed that several IRXs, GUXs, GXMTs, and XATs were abundantly present and matched their corresponding transcript profiles (**Figure 2**; **Supplementary Data Sheet 5**). Also in our study, the transcript levels of *GUXs* and *GXMTs*, were found to be higher in *Gb* than in *Gh*. Additional research is still required to understand the temporal expression dynamics of IRXs, GUXs, GXMTs, XATs and heteroxylans accumulation in fiber and their impact on fiber quality. Interestingly, an earlier study of *Arabidopsis* mutants showing various levels of glucuronoxylan deficiency demonstrated that the levels of glucuronoxylans critical for the precise cellulose network formation and CW integrity of SCWs (Crowe et al., 2021). This might suggest that temporal synchrony in the presence of appropriate heteroxylans quality and quantity in combination with the other polysaccharides, affects the CW architecture and, thus, the final quality of the fiber.

Pectins are the major components of the PCW and CFML (up to 35% dry weight) of the cotton fiber (Kaczmarska et al., 2022). There are four main structural components of pectins: HGs, RG-I, rhamnogalacturonan-II, and xylogalacturonans. The cellulose and hemicellulose networks are embedded in a matrix of pectins and proteins in the PCW. HGs and RGs play a central role in regulating the viscosity/extensibility/gelling property of the CW matrix, thus controlling polysaccharide interactions, CW elongation, and cell growth (Hwang and Kokini, 1992; Yapo, 2011; Sousa et al., 2015; Zheng et al., 2020). Our results from glycome profiling showed that a methyl-esterified HG epitope (HG-BBMe-K) and most of the highly branched RG-I epitopes were less abundant in *Gb* relative to *Gh*. In contrast, the de-esterified HGs (HG-BBde), and RG-I backbone epitopes (RG-I-BB, Gal4-BB) were present in equal amounts in both species. Similar observations were reported earlier by Avci et al. (2013). Another comparative “omics” study (Liu et al., 2013) reported a lower amount of methyl-esterified HG in *Gb* compared to *Gh* during elongation time and proposed that pectin content might be an important factor influencing fiber elongation and quality. Our results suggest that the presence of a lower content of HGs and highly branched RG-Is, in addition to the changes in other polysaccharide epitopes during the PCW developmental/fiber elongation stage in *Gb*, could support its longer fiber phenotype.

Our comparative transcript analysis showed that transcript levels of most of the glycosyltransferases (that synthesize the HG epitopes), including methyl and acetyltransferases (*GAUT*s, *GATL*s, *CGR*s/*QUA*s, *TBL*s/*TBR*s) were present in higher amounts in *Gh* than in *Gb*. A similar trend was observed for RG-I-synthesizing glycosyltransferases, most likely resulting in a higher amount of pectin epitopes in *Gh* than in *Gb*. The cotton AG: GALT (Gohir.A04G041100/*Gh*AG: GALT1-2At) protein was found to play a functional role in galactosylation of AG chains of RG-I/AGPs molecules (Qin et al., 2017). The *AG: GALT1-2At* RNAi-silenced cotton plants, had less galactose content in the RG-I and produced longer fibers as a result of CW loosening. Conversely, AG: GALT1-2At protein overexpressing cotton plants had the opposite effect. Overexpression of an HG- degrading polygalacturonase in *Arabidopsis* resulted in reduced HG content, which resulted in a longer hypocotyl and this might be because of increased CW loosening (Xiao et al., 2014). Pectate lyases (PEL) are another type of hydrolase involved in the degradation of HGs. Silencing of a cotton *GhPEL* resulted in accumulation of higher amounts of HGs in fiber CW during the elongation stage, which subsequently resulted in a shorter fiber phenotype in transgenic plants (Wang et al., 2010).

All these results indicate that higher amounts of pectin epitopes during the elongation stage lead to possible broader hydrogen bonding interactions (as reported in *in vitro* studies; Zheng et al., 2020; Chandel et al., 2022; Said et al., 2023) or calcium-mediated “egg-box” formations (Cosgrove, 2022) between the pectin backbone molecules, both can lead to increased firmness of CW, which could negatively impact the elongation time and length of cotton fibers. A more detailed comparative investigation of glycosyl hydrolases contributing to cotton fiber development will be conducted in our future study.

### 4.3 Differentially accumulated transcript levels of expansins of *Gh* and *Gb* fibers

Expansins are CW-loosening proteins that act in a non-enzymatic manner to weaken the noncovalent bonds in hemicellulose-cellulose networks, resulting in slippage of cellulose microfibrils, and ultimately leading to cell expansion (Sampedro and Cosgrove, 2005) and one of the major factors responsible for cotton fiber elongation (Li et al., 2016; Lv et al., 2020). Therefore, here, in addition to glycosyltransferases, we examined the transcript levels of known expansins in both *Gh* and *Gb* fibers. Interestingly, the transcript levels of most of the expansins was higher in *Gb* relative to *Gh* (**Figure 13**; **Supplementary Data Sheet 9**). Additionally, the transcript levels of some of the expansins was significantly higher in *Gb* through the later stages (after 17 DPA), which coincides with the extended elongation stage in this cotton genotype. In contrast, in *Gh*, the transcript levels of most expansins decreased at 17 DPA, coinciding with the transition stage before SCW thickening and complete cessation of fiber elongation in this genotype. Our results suggest that the temporally controlled higher level of expansin expression plays an important role in extended fiber elongation and the development of superior fiber quality in *Gb*.

Previous reverse-genetics studies of *EXPA8-2* (expansin with the highest transcript accumulation levels from both *Gh* and *Gb*) by Xu et al. (2013) and Li et al. (2016) showed that the cotton fiber length is increased in *Gb*EXPA8–2 overexpressing lines and reduced in the silenced lines. Overexpression of *Gb*EXPA8–2 protein also altered the expression of SCW-associated genes and delayed the accumulation of SCW cellulose, which resulted in extended elongation time of the PCW stage and long fibers in *Gb*. A separate transgenic experiment in *Gh* by overexpressing *Gh*EXPA8–2 protein along with a BURP-domain containing protein resulted in the increased fiber length (Xu et al., 2013).

## 5 Conclusion

**Figure 14:**
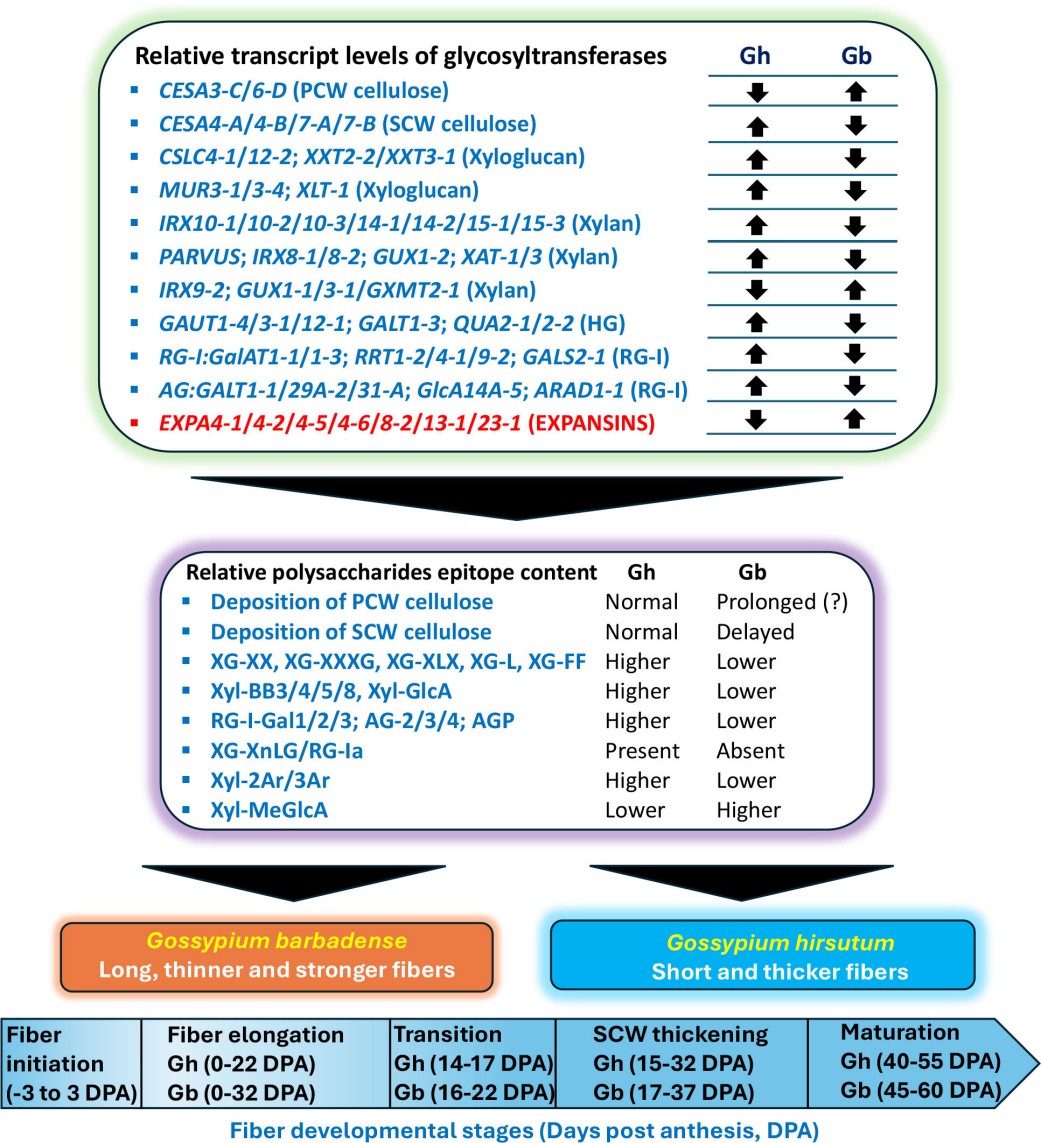
The chart summarizes the key differences in polysaccharide composition and glycosyltransferasesof *Gh* and *Gb* fibers found in this study. The differences observedin the transcript levels of various glycosyltransferases and expansins are a potential cause of the differential polysaccharide epitope contents during fiber development (**Supplementary Data Sheet 8**). We propose that these differences might contribute to the differences in fiber qualitybetween the two cotton species. Upward and downward arrows indicate the relative higher and lowerlevels of accumulated glycosyltransferase transcripts in *Gh* and *Gb* fibers (**Supplementary Data Sheet 4**).

In summary, this study integrated large-scale CW polysaccharide glycome profiling and glycosyltransferase transcriptome profiling of the two commercially important cotton species*, Gb* and *Gh*, which have distinct fiber qualities. The main focus of our study was to elucidate the roles of potential polysaccharide epitopes and their temporal dynamics in determining fiber quality between the two cotton species through a comparative analysis. In addition, we attempted to compare transcript levels of known glycosyl transferases using bioinformatics. Our comparative study at high temporal resolution identified potentially critical polysaccharide epitopes and polysaccharide-synthesizing glycosyltransferases that may determine or contribute to the differences in final fiber quality and characteristics in cotton (**Figure 14**). The presence of relatively lower amounts of methyl esterified HG, highly branched RG-I epitopes, XG, and Xyl epitopes most likely impacted both the length and strength of cotton fiber by extending fiber elongation time in *Gb* compared to *Gh*. Higher and prolonged expression of PCW CESAs, delayed expression of SCW CESAs and corresponding delayed cellulose accumulation that might have contributed to the longer fiber phenotype of *Gb*. Additionally, our study suggests that the differentially accumulated heteroxylans (Xyl-2Ar, Xyl-3Ar and Xyl-MeGlcA), might also be important for cellulose microfibril arrangement and different strengths of the fibers in both species. Our study, together with multiple earlier reports, identified potential polysaccharide structures and associated glycosyltransferases that contribute to the differences in fiber quality (**Figure 14**). Recently, the prospects of using molecular genetics to engineer cotton fiber phenotypes have shown promise. Therefore, the newly obtained insights into the molecular control of fiber morphology and quality will be a valuable source of information for future steps in not only refining the specific factors dictating the final quality of cotton fiber but also providing directions for quality improvement through cotton transformation.

## Data Availability

RNAseq reads of cotton fibers collected from 6 to 25 DPA were deposited into the NCBI-SRA under PRJNA1099209 (Gossypium hirsutum) and PRJNA1222456 (Gossypium barbadense). All the data and materials that support the findings of this study are available upon request from the corresponding author.

## Supplementary material

Supplementary Data Sheet 1 Fiber cell wall polysaccharides of *Gossypium barbadense* and *Gossypium hirsutum* (6–25 DPA).

Supplementary Data Sheet 2 List of polysaccharide specific antibodies used in this study and their binding epitopes.

Supplementary Data Sheet 3 Glycome profiling data of *Gossypium barbadense* and *Gossypium hirsutum* (6–25 DPA).

Supplementary Data Sheet 4 List of cell wall polysaccharide synthesizing enzymes from *Arabidopsis* and cotton.

Supplementary Data Sheet 5 Proteome data and correlation analysis of transcriptomic and proteomic profiling data of glycosyltransferases during cotton fiber development.

Supplementary Data Sheet 6 Pearson Correlation Coefficient (PCC) analysis of *Gossypium barbadense* glycome, proteome and transcriptome profiles data.

Supplementary Data Sheet 7 Self-organizing map (SOM) groups of glycome profiled polysaccharide fractions and the epitopes present in each group.

Supplementary Data Sheet 8 Categories of polysaccharides epitopes of *Gossypium barbadense* and *Gossypium hirsutum* (6–25 DPA) based on SOM.

Supplementary Data Sheet 9 List of cotton expansins genes homologous to genes of *Arabidopsis* expansins.
